# Piperacetazine Directly Binds to the PAX3::FOXO1 Fusion Protein and Inhibits Its Transcriptional Activity

**DOI:** 10.1158/2767-9764.CRC-23-0119

**Published:** 2023-10-06

**Authors:** Kay Nakazawa, Taryn Shaw, Young K. Song, Marilyn Kouassi-Brou, Anna Molotkova, Purushottam B. Tiwari, Hsien-Chao Chou, Xinyu Wen, Jun S. Wei, Emre Deniz, Jeffrey A. Toretsky, Charles Keller, Frederic G. Barr, Javed Khan, Aykut Üren

**Affiliations:** 1 Department of Oncology, Georgetown University Medical Center, Georgetown University, Washington, District of Columbia.; 2 Genetics Branch, Center for Cancer Research, NCI, NIH, Bethesda, Maryland.; 3 Children's Cancer Therapy Development Institute, Hillsboro, Oregon.; 4 Laboratory of Pathology, Center for Cancer Research, NCI, Bethesda, Maryland.

## Abstract

The tumor-specific chromosomal translocation product, PAX3::FOXO1, is an aberrant fusion protein that plays a key role for oncogenesis in the alveolar subtype of rhabdomyosarcoma (RMS). PAX3::FOXO1 represents a validated molecular target for alveolar RMS and successful inhibition of its oncogenic activity is likely to have significant clinical applications. Even though several PAX3::FOXO1 function-based screening studies have been successfully completed, a directly binding small-molecule inhibitor of PAX3::FOXO1 has not been reported. Therefore, we screened small-molecule libraries to identify compounds that were capable of directly binding to PAX3::FOXO1 protein using surface plasmon resonance technology. Compounds that directly bound to PAX3::FOXO1 were further evaluated in secondary transcriptional activation assays. We discovered that piperacetazine can directly bind to PAX3::FOXO1 protein and inhibit fusion protein-derived transcription in multiple alveolar RMS cell lines. Piperacetazine inhibited anchorage-independent growth of fusion-positive alveolar RMS cells but not embryonal RMS cells. On the basis of our findings, piperacetazine is a molecular scaffold upon which derivatives could be developed as specific inhibitors of PAX3::FOXO1. These novel inhibitors could potentially be evaluated in future clinical trials for recurrent or metastatic alveolar RMS as novel targeted therapy options.

**Significance:**

RMS is a malignant soft-tissue tumor mainly affecting the pediatric population. A subgroup of RMS with worse prognosis harbors a unique chromosomal translocation creating an oncogenic fusion protein, PAX3::FOXO1. We identified piperacetazine as a direct inhibitor of PAX3::FOXO1, which may provide a scaffold for designing RMS-specific targeted therapy.

## Introduction

Rhabdomyosarcoma (RMS) is the most common soft-tissue sarcoma in children and adolescents, accounting for nearly half of all pediatric soft-tissue sarcomas (1). There are two major histologically distinct subtypes of pediatric RMS: embryonal rhabdomyosarcoma (ERMS) and alveolar rhabdomyosarcoma (ARMS), with ARMS typically resulting in a lower survival rate (1–3). RMS cells express skeletal muscle-specific markers such as MYOD1, myogenin (MYOG), myoglobin (MB), desmin (DES), and muscle-specific actin (ACTA1), whose expression can be used for diagnostic purposes (4–8). The current standard of care includes a combination of surgery, radiation, and chemotherapy, depending on tumor size and location. Combination chemotherapy with vincristine, actinomycin-D, and cyclophosphamide has been the standard of care for the past 40 years without the successful introduction of any novel targeted therapy except temsirolimus (9, 10).

Approximately 80% of ARMS cases are characterized by the tumor-specific chromosomal translocations [t(2;13) or t(1;13)] that generate a *PAX3::FOXO1* or a *PAX7::FOXO1* gene fusion (2, 11, 12). Of the two fusion proteins, PAX3::FOXO1 is the more common, occurring in approximately 70% of fusion-positive RMS (FP-RMS; ref. 13). The PAX3::FOXO1 and PAX7::FOXO1 fusion proteins retain the DNA-binding domains of the PAX3 and PAX7 proteins and the transcriptional activation domain of FOXO1, and are 10- to 100-fold more transcriptionally active at PAX3/7 DNA-binding sites than either wild-type PAX3 or PAX7 (11, 12, 14–16). Expression of PAX3::FOXO1 in a regulatable myoblast model causes colony formation *in vitro* and tumor formation *in vivo*, while reduced fusion protein expression inhibits both phenotypes (17). PAX3::FOXO1 reduction sensitizes tumors to radiation in a xenograft model (18). Patients with FP-RMS tumors have a lower survival rate than those with fusion-negative RMS (FN-RMS; refs. 19, 20). Therefore, these oncogenic fusion proteins are important factors for initiation and maintenance of RMS and can be exploited for therapeutic purposes.

The PAX3::FOXO1 fusion protein is a key regulator of FP-RMS oncogenesis and its unique expression in tumor tissues supports development of direct inhibitors. In recent years, several drugs that inhibit PAX3::FOXO1-responsive reporter constructs or phenocopy the loss of the PAX3::FOXO1 gene expression profile have been reported, including thapsigargin, entinostat, fenretinide, JQ-1, SAHA, and tideglusib (21–26). Other molecules tested in RMS models target PAX3::FOXO1-activated signaling cascades, including rapamycin and ponatinib (27, 28). However, none of these drugs directly bind to PAX3::FOXO1 to specifically inhibit its function. Therefore, the empiric discovery of direct inhibitors of the PAX3::FOXO1 protein should be considered.

In the current study, we identify a small molecule, piperacetazine, that directly binds to PAX3::FOXO1. Piperacetazine was discovered in the 1960s and approved by the FDA for the treatment of schizophrenia as a phenothiazine derivative that acts via dopamine D_2_ receptor (DRD2) antagonism (29, 30). Our research shows that piperacetazine is an inhibitor of PAX3::FOXO1 transcriptional activity and also inhibits FP-RMS anchorage-independent growth. We propose piperacetazine as a novel scaffold for derivative development toward future new agents in treating FP-RMS.

## Materials and Methods

### Chemicals

Unless stated otherwise, all chemicals and reagents were purchased from MilliporeSigma. Compound libraries for screening were obtained from the Developmental Therapeutics Program (RRID:SCR_003057) of the NCI and from the Prestwick Chemicals. The following compounds selected for further study were commercially obtained: carboplatin and didanosine (SelleckChem catalog nos. S1215 and S1702), alcuronium chloride (MilliporeSigma catalog no. 15180-03-07), piperacetazine (MedChemExpress catalog no. HY-B1152), and NSC2805 (Glixx Labs).

### PAX3::FOXO1 Purification

Full-length *PAX3::FOXO1* (GenBank accession code: AAC50053.1) was cloned into pET104.1 DEST plasmid with a carboxy terminal 12 His tag and an amino terminal internal HA tag (Supplementary Fig. S1). Recombinant protein expression was induced by isopropyl-β-D-thiogalactopyranoside in *Escherichia coli* strain BL-21 (DE3). Cell lysates were prepared using the BugBuster MasterMix (MilliporeSigma catalog no. 71456-4). Purification was done using a HiTrap Chelating HP 1 mL column in AKTA Pure 25 Explorer (Cytiva). The column was washed with water, charged with 100 mmol/L nickel sulfate and washed again with water. The cell pellet was dissolved in the running buffer (20 mmol/L sodium phosphate buffer pH 7.4, containing 500 mmol/L NaCl, 40 mmol/L imidazole), which was also used for equilibrating the column before sample application and consequent wash. Recombinant protein was eluted from the column with a linear gradient to running buffer containing 1 mol/L imidazole.

### Surface Plasmon Resonance (SPR)

Recombinant PAX3::FOXO1 and negative control CD99 proteins were immobilized onto a Biacore CM5 sensor chip by amine coupling in a Biacore 4000 instrument (Cytiva Life Sciences, RRID:SCR_023766). Proteins were immobilized on the same CM5 chip in neighboring spots of the same flow cell at two different surface densities. High-density (HD) PAX3::FOXO1 surfaces had 2,700–4,200 RU protein. Low-density (LD) PAX3::FOXO1 surfaces had 400–700 RU protein. HD CD99 surfaces had 1,800–3,200 RU protein. LD CD99 surfaces had 500–800 RU protein. The theoretical binding capacity of R_max_ was calculated for each compound based on the amount of ligand captured on each spot and the molecular weights of each compound (R_max_ = R_L_ × MW of analyte/MW of ligand). R_max_ calculations were made with a 1:1 binding expectation. Compounds were injected individually at 50 µmol/L, and the binding sensorgrams were recorded. We used a double reference subtraction method to eliminate any bulk refractive index contribution to the measured binding signal, which was achieved by having an empty reference spot without any protein in all four flow cells of the CM5 chip and by injecting buffer alone over all surfaces. The signal coming from the empty reference spots and the buffer alone injections were subtracted from all compound signals to account for background noise. A solvent correction was applied to the sensorgrams to account for possible minor mismatch in DMSO content in between running buffer and small-molecule dilutions in the running buffer. Compounds were eliminated from further consideration if they (i) gave a higher signal on LD surface than HD surface for PAX3::FOXO1, (ii) bound to the HD PAX3::FOXO1 surface with greater than 200% of the expected R_max_ value, or (iii) bound to the HD PAX3::FOXO1 surface with less than 50% of the expected R_max_ value. The remaining compounds were compared for their binding signal on an HD PAX3::FOXO1 surface with an HD CD99 surface. Because the expected R_max_ value for each analyte is proportional to the molecular weight ratios and the surface density, the binding values were first normalized and expressed as % of R_max_. Then, the % of R_max_ of PAX3::FOXO1 binding was divided by % of R_max_ of CD99 binding, and the compounds were ranked on the basis of this fold ratio. Any compound that showed 5-fold or higher binding to PAX3::FOXO1 was considered a primary hit. Validation binding experiments with small molecules and DNA were done in a Biacore T-200 system (RRID:SCR_019718). PAX3::FOXO1 and EWS::FLI1 were immobilized on CM5 chips via standard amine coupling reaction. PBS-P (20 mmol/L phosphate buffer at pH 7.4, 137 mmol/L NaCl, 2.7 mmol/L KCl, 0.05% v/v surfactant P20) was used as the immobilization running buffer. For small-molecule studies, PBS-P was supplemented with 5% v/v DMSO. The following oligonucleotides were used in SPR studies:

PAX3::FOXO1:

- 5′-ACCGTGACTAATTTAATTAGTCACG-3′
- 5′-CGTGACTAATTAAATTAGTCACGGT-3′

EWS::FLI1:

- 5′-ACCGGAAGGAAGGAAGGAAGGAAGGAAGGAAGTG-3′
- 5′-TGGCCTTCCTTCCTTCCTTCCTTCCTTCCTTCAC-3′

### CETSA

Collected cell lysates were treated with 30 µmol/L piperacetazine or DMSO (negative control) for 30 minutes at room temperature and heated for 3 minutes at the indicated temperatures, followed by a 3-minute incubation at room temperature. The samples were then centrifuged at 16,000 × *g* for 30 minutes at 4°C. Protein levels in the remaining supernatant were evaluated by immunoblotting. Quantification was done using Image Studio Lite (LI-COR Biosciences, RRID:SCR_013715).

### Cell Culture

Murine U66788, U48484, U37125, and U57810 RMS cells were originally established from transgenic mouse models of ARMS and ERMS and grown in DMEM supplemented with 10% FBS (31–34). The RH28 cell line used in this study was a subclone selected for resistance to L-PAM (RRID:CVCL_Y584). RH28, RD (ATCC catalog no. CCL-136, RRID:CVCL_1649), HEK293T (ATCC catalog no. CCL-3216, RRID:CVCL_0063), K7M2 (RRID:CVCL_V455), K12 (RRID:CVCL_W625), U2OS (RRID:CVCL_0042), MG63.3 (RRID:CVCL_WL01), HeLa (ATCC catalog no. CCL-2, RRID:CVCL_0030), and SN12C cells (RRID:CVCL_1705) were grown in DMEM supplemented with 10% FBS. STA-ET-7.2 (RRID: CVCL_9693), RH30 (ATCC catalog no. CRL-2061, RRID:CVCL_0041), and RH41 (RRID:CVCL_2176) cells were grown in RPMI supplemented with 10% FBS, as well as A4573 (RRID:CVCL_6245), which was additionally supplemented with 1% HEPES. Dbt-MYCN/indP3F cells were grown in Ham's/F-10 medium supplemented with 15% FBS, 50 µg/mL uridine, 1 mmol/L sodium pyruvate, and 1 mmol/L creatine. Cell lines were routinely tested for the presence of *Mycoplasma*, with the latest test on July 6, 2023, using the MycoAlert system according to manufacturer's instructions (Lonza catalog no. LT07-318) and fingerprinted for identity confirmation (Supplementary Table S1).

RH28 and RH41 cell lines were kindly provided by Dr. Peter Houghton, University of Texas Health San Antonio (San Antonio, TX). K7M2, K12, U2OS, and MG63.3 cell lines were kindly provided by Dr. Chand Khanna, NIH. SN12C cells were kindly provided by Dr. Chunling Yi, Georgetown University. STA-ET-7.2 cells were kindly provided by Dr. Heinrich Kovar, Children's Cancer Research Institute. A4573 cells were kindly provided by Dr. Tim Triche, Children's Hospital Los Angeles. Stable RH30 cells expressing the PAX3::FOXO1-responsive 6XPRS-luciferase reporter were kindly provided by Dr. Asoke Mal, Roswell Park Cancer Center. RH30, HEK293T, HeLa, and RD cells were purchased from ATCC.

### Luciferase Reporter Assays

Stable RH30 PAX3::FOXO1-responsive 6XPRS-Luc and SN12C phosphoglycerate kinase (PGK)-responsive luciferase reporter cells were seeded in 96-well plates at 10,000 cells per well and allowed to grow for 24 hours. Cells were treated with test compounds at a concentration of 10 µmol/L in DMSO. Luciferase activity was measured after 48 hours of treatment. In a separate assay, cell lines were transfected with a pGL3-*PDGFRA* PAX3::FOXO1-responsive luciferase construct using X-tremeGENE 9 DNA Transfection Reagent (Roche catalog no. XTG9-RO) 24 hours prior to seeding, then seeded in 96-well plates at 5,000–40,000 cells per well and treated with piperacetazine or vehicle control. Luciferase activity was measured after 48 hours of drug treatment. HEK293T cells (RRID:CVCL_0063) were used in a third luciferase assay, in which cells were transfected with PAX3::FOXO1-responsive pGL4.19-*ASS1P* luciferase reporter and either an empty vector or *PAX3::FOXO1* construct, then treated with DMSO or piperacetazine. After 48 hours, the cells were lysed for the luciferase assay, and the same number of cells were lysed for protein quantification via Pierce bicinchoninic acid assay (Thermo Fisher Scientific catalog no. 23225). The luciferase assay readouts were then normalized to the total protein amounts. In all experiments, luciferase activity was quantified using a luciferase assay system (Promega catalog no. E1501) according to manufacturer's instructions.

### Immunoblotting

Immunoblotting was performed using anti-FOXO1 (Cell Signaling Technology catalog no. 2880, RRID:AB_2106495), MYOD1 (Cell Signaling Technology catalog no. 13812, RRID:AB_2798320), MYOG (Novus catalog no. NB100-56510, RRID:AB_838604), B7-H3 (CD276) (Cell Signaling Technology catalog no. 14058, RRID:AB_2750877), PAX3 (Cell Signaling Technology catalog no. 12412, RRID:AB_2636922), phospho-FOXO1 (pSer256; Cell Signaling Technology catalog no. 9461, RRID:AB_329831), and actin-HRP (Abcam catalog no. ab20272, RRID:AB_445482) antibodies. Whole-cell lysates from cells grown to near confluence were subjected to SDS-PAGE and transferred to a Low Fluorescence polyvinylidene difluoride Membrane (Bio-Rad catalog no. 1704275). The membranes were then subject to blocking in 5% nonfat dry milk in 1 × TTBS (Tween-Tris-buffered saline; 20 mmol/L Tris-HCl, pH 7.5, 150 mmol/L NaCl, 0.5% Tween 20) for 1 hour. Primary antibodies were added to the membrane in 5% BSA in 1 × TTBS for 2 hours. The membrane was then washed three times in 1 × TTBS, and horseradish peroxidase–linked anti-rabbit or anti-mouse secondary antibody (Thermo Fisher Scientific catalog no. NA934; catalog no. NA9311ML) in 5% nonfat dry milk was added for 1 hour. The blots were then washed three times in 1 × TTBS and developed using Millipore Immobilon Western chemiluminescent horseradish peroxidase substrate per the manufacturer's instructions (Millipore catalog no. WBKLS0500). Chemiluminescence was detected using a LI-COR Odyssey Fc imaging system (RRID:SCR_023227). Densitometry values were generated using Image Studio Lite software.

### Cell Viability Assays

Cells were seeded on 96-well plates at a density of 5,000–10,000 cells/well in 100 µL of media. Piperacetazine or vehicle control were added after 24 hours, and cell viability was measured at 48 hours posttreatment using the CellTiter-Blue assay and measuring fluorescence following manufacturer's instructions (Promega catalog no. G808A) on a BioTek Synergy H4 plate reader (RRID:SCR_019750).

### siRNA Transfection and Cell Proliferation Assays

Real-time electrical impedance data were collected using an xCelligence RTCA DP instrument (Agilent Part no. 00380601050). RH30 and U66788 cells were transiently transfected with a custom *PAX3::FOXO1* siRNA (5′-GCCUCUCACCUCAGAAUUCtt-3′; Thermo Fisher Scientific catalog no. 4390828) using X-TremeGENE siRNA transfection reagent for 24 hours before being plated in an xCelligence RTCA 16-well e-plate. Prior to cell seeding, the wells of the e-plates were coated with collagen for 1 hour at 37°C and washed with PBS. Media was added to each well for an equilibration measurement, and the cells were added to the wells (5,000 cells/well). The remaining cells were collected and lysed for Western blot analysis. RTCA software (RRID:SCR_014821) was used to collect impedance data from the e-plate at 10-minute intervals for 48 hours, which was graphed using GraphPad Prism software (RRID:SCR_002798).

### RNA Sequencing and Gene Set Enrichment Analysis

RH30 cells were treated with 30 µmol/L of piperacetazine or DMSO for 24 hours before harvest for RNA using RNAeasy kits (Qiagen catalog no. 74104). RNA sequencing (RNA-seq) libraries were constructed using Illumina TruSeq stranded mRNA sample preparation kits and sequenced on a NextSeq500 sequencer using 2 × 75 bp paired-end protocol (Illumina). Genes with >10 total reads in these samples were kept, and raw read counts were transformed to regularized logarithm values using DESeq2 package (RRID:SCR_015687; ref. 35). The ranked list of genes for the comparison between piperacetazine-treated and DMSO control were sorted by log_2_ fold change. The gene set enrichment analysis (GSEA) was performed using command “gsea-cli.sh GSEAPreranked” and curated gene sets (36).

### Synergy Experiments

RH30 and RD cells were plated in 384-well plates at a seeding density of 2,000 cells/well and placed in the 37°C incubator overnight. The following day, drug dilutions were prepared for piperacetazine and actinomycin D, JQ-1, vincristine, or entinostat, and combinations of concentrations from each drug were prepared in a 96-well plate and then transferred to the wells. A total of 48 hours posttreatment, CellTiter-Blue reagent was added to the wells, and the plates were incubated for 2–3 hours at 37°C before fluorescence measurements were taken according to the manufacturer's protocol on a BioTek Synergy H4 plate reader (RRID:SCR_019750). The results were analyzed via the HSA, Bliss, Loewe, and ZIP synergy models using SynergyFinder software (RRID:SCR_019318; ref. 37).

### Soft Agar Assays

A total of 0.6% agar in cell culture media was prepared for the bottom layer of the wells of a 24-well plate and 1 mL of this mixture was distributed to each well. Once solidified, RD and RH30 cells were seeded in 500 µL of a 0.4% agar top layer containing 15 µmol/L piperacetazine or DMSO at a concentration of 5,000 cells/well. After this top layer solidified, 75 µL of 15 µmol/L piperacetazine or DMSO in media was added to the wells before the plate was placed in the 37°C incubator. The wells were fed one to two times weekly with 15 µmol/L piperacetazine or DMSO. Plate images were taken at day 19 postseeding on a GelCount colony counter (RRID:SCR_023219) and quantified using the accompanying software. Individual well images were taken using a Nikon Ti-Eclipse inverted microscope at a 40x magnification (RRID:SCR_021242).

### Mouse Xenograft Studies

All animal studies were conducted under a protocol approved (approval number 2019-0068) by the Georgetown University's Institutional Animal Care and Use Committee in accordance with NIH guidelines for the ethical treatment of animals. NOD-SCID mice (Charles River Laboratories, RRID:IMSR_CRL:394) of both sexes were used for all xenograft studies. Animals receiving RH30 xenografts were fed regular diet. The group that was scheduled to receive Dbt-MYCN/indP3F injections began a 625 mg/kg doxycycline diet (Envigo Teklad). After 3 days, mice were injected in the left gastrocnemius muscle with RH30 (2 × 10^6^ cells /100 µL PBS) or Dbt-MYCN/indP3F (1 × 10^6^ cells/100 µL PBS) cells. After primary tumors reached 200–250 mm^3^ in size, mice were randomly allocated to vehicle control (DMSO) or piperacetazine treatment groups. For Dbt-MYCN/indP3F mice, a third group was assigned to vehicle control and doxycycline feed withdrawal. For drug treatment, piperacetazine was solubilized in DMSO as a 40 mg/mL solution for intraperitoneal administration, and the dosing solutions were prepared by 10x dilution in sterile PBS. Mice received approximately 0.1 mL injections of this solution or vehicle [DMSO, 10% (v/v)]. Doses were administered 5 days per week for the indicated duration of time. Tumors were measured 5 days per week using slide calipers, and tumor volumes were determined by the formula length × width^2^/6 × 3.14. Animals were euthanized when tumor volume reached 1.5 cm^3^. For RH30 xenograft experiments, AUC comparisons were performed as described previously (38).

### Statistical Analysis

Statistical analysis was performed using GraphPad Prism version 9.0 (RRID:SCR_002798). Statistical significance was defined as *P* < 0.05.

### Data Availability Statement

The data generated in this study are available within the article and its Supplementary Data. RNA-seq data are available through Gene Expression Omnibus database (accession number: GSE242690).

## Results

### Small Molecules Binding to PAX3::FOXO1 Protein were Identified by SPR

**FIGURE 1:**
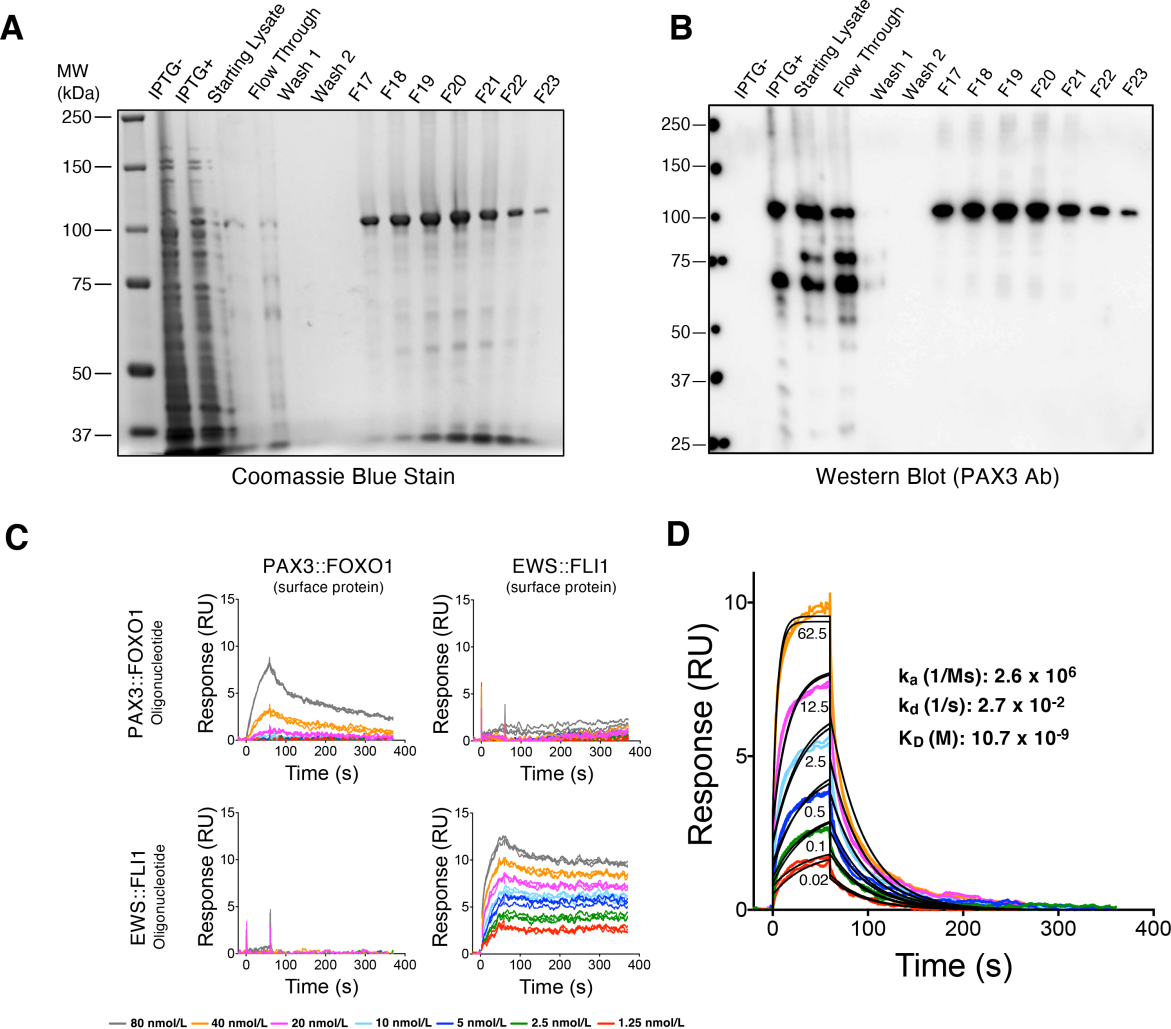
Purification of recombinant PAX3::FOXO1 protein from bacteria. **A,** PAX3::FOXO1 protein is expressed in *E. coli*. The most prominent bands following induction with isopropyl-beta-D-thiogalactopyranoside (IPTG) appeared just above the 100 kDa molecular weight marker on an 8% polyacrylamide gel stained with Coomassie blue. F17–F23 represent seven different fractions purified by column chromatography using the His tag on the carboxy terminal of the protein. **B,** The same purified fractions in A were used in a Western blot analysis to confirm protein identity using an anti-PAX3 antibody. **C,** DNA-binding specificity was evaluated by measuring binding of recombinant PAX3::FOXO1 and EWS::FLI1 proteins to double-stranded DNA oligonucleotides with binding sequences specific to either protein. PAX3::FOXO1 or EWS::FLI1 protein was immobilized on a CM5 chip and the indicated oligonucleotides were injected over the surface at a 1.25 to 80 nmol/L dose range. **D,** The quality of the PAX3::FOXO1 protein was evaluated by measuring its binding affinity to a double-stranded DNA oligonucleotide. PAX3::FOXO1 was immobilized on a CM5 chip and the oligonucleotide was injected over the surface at six concentrations (62.5, 12.5, 2.5, 0.5, 0.1, 0.02 nmol/L). Binding affinity was calculated by using a 1:1 binding model in BiaEvaluation software (*χ*^2^: 0.0424, *U*-value: 2). Colored lines indicate actual datapoints and black lines indicate curve fit used for affinity analysis.

To screen for small-molecule binders of PAX3::FOXO1 protein, we began by expressing recombinant PAX3::FOXO1 in bacteria and purifying the protein via column chromatography. We purified recombinant PAX3::FOXO1 protein to >90% purity at 50 µg/mL concentration (Fig. 1A). The identity of the recombinant PAX3::FOXO1 protein was confirmed by Western blot analysis using an anti-PAX3 antibody (Fig. 1B). We confirmed DNA-binding specificity of the purified protein by comparing its binding with oligonucleotides specific for PAX3::FOXO1 or EWS::FLI1 (a tumor-specific fusion oncoprotein found in Ewing sarcoma) using SPR. Purified PAX3::FOXO1 and EWS::FLI1 proteins were immobilized on neighboring flow cells of a CM5 chip by amine coupling and fusion protein–specific DNA oligonucleotides were injected over both protein-coated surfaces at a range of concentrations while the interactions were measured in real time. PAX3::FOXO1 protein bound to a PAX3::FOXO1-specific oligonucleotide but not an EWS::FLI1-specific one, while the reverse was true of EWS::FLI1 protein (Fig. 1C). We further derived the kinetics parameters of the purified recombinant PAX3::FOXO1 protein binding to its specific double-stranded DNA oligonucleotide. A representative result is provided in Fig. 1D. The experiment was repeated six times, yielding an average K_D_ value of 6.3 nmol/L (SE 2.3 nmol/L). Therefore, we concluded that the recombinant protein had a properly folded DNA binding domain and was deemed to be suitable for small-molecule screening experiments.

**FIGURE 2:**
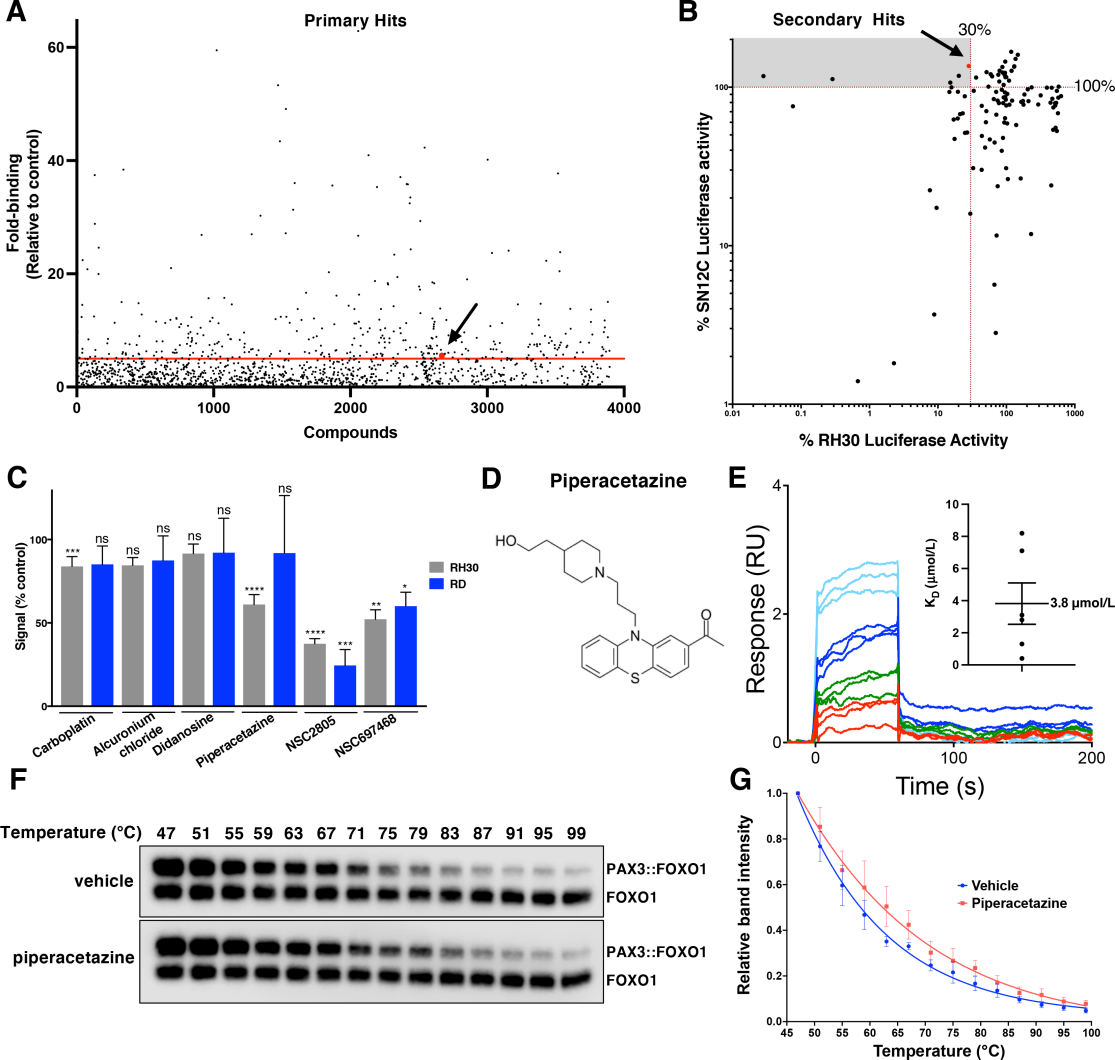
Screening of small-molecule libraries for PAX3::FOXO1 binding. **A,** Binding of individual compounds from Prestwick or DTP libraries is shown as fold difference of PAX3::FOXO1 binding compared with the negative control protein (CD99). Each dot represents an individual compound. The red line indicates the 5-fold binding threshold used for the selection of primary hits. Piperacetazine is labeled in red. **B,** Inhibition of luciferase activity in fusion-positive RMS cell line RH30 with PAX3::FOXO1-responsive 6XPRS reporter and renal cell carcinoma cell line SN12C with PGK reporter (negative control). Responses are normalized to DMSO control. Compounds in the top left quadrant are considered potential PAX3::FOXO1 inhibitors, piperacetazine is labeled in red. **C,** PAX3::FOXO1-responsive *PDGFRA* luciferase reporter was transfected into RH30 and RD cell lines and treated with the top six hits identified by the secondary screening as shown in B. The cells were lysed for luciferase assay 48 hours posttreatment, and the luciferase signal was quantified. (****, *P* < 0.0001; ***, *P* < 0.001; **, *P* < 0.01; *, *P* < 0.05; Student *t* test; ns, *P* > 0.05). **D,** The structure of piperacetazine is given. **E,** Direct binding of piperacetazine to PAX3::FOXO1 protein was analyzed by SPR. Piperacetazine was injected at 1.25, 2.5, 5, and 10 µmol/L concentrations in triplicates. A representative experiment is shown. An average K_D_ of 3.8 µmol/L was calculated from six experiments (Inset). **F,** RH30 cells lysates were incubated with 30 µmol/L piperacetazine or DMSO and heated to the indicated temperatures. Lysates were centrifuged to separate precipitated proteins and soluble protein levels were measured by Western blot analysis using FOXO1 antibody (in each blot, top band: PAX3::FOXO1, bottom band: FOXO1). **G,** Quantification of the three replicate experiments is provided in a line graph by plotting mean and SE values.

SPR was used for screening two small-molecule libraries in a Biacore 4000 instrument. We screened 3,894 compounds covering a wide spectrum of structure classes from the Prestwick Chemical Company and the Developmental Therapeutics Program of the NCI (diversity set, mechanistic set, natural products set). The binding of each compound to recombinant PAX3::FOXO1 protein was compared with the binding to another intrinsically disordered recombinant protein with a carboxy terminal His tag, CD99, which was used as a negative control. On the basis of a stringent hit selection criteria as explained in the Materials and Methods section, we selected 119 compounds capable of binding to PAX3::FOXO1, which were advanced to a secondary screening assay based on PAX3::FOXO1 activity (Fig. 2A; Supplementary Table S2).

The secondary screen that evaluated inhibition of PAX3::FOXO1 transcription initiation used the FP-RMS cell line RH30. PAX3::FOXO1 activates 6XPRS promoter luciferase construct allowing for easy quantification of its function (39). As a negative control, we used the renal cell carcinoma cell line SN12C that was stably transfected with a human PGK promoter luciferase construct. Both cell lines were treated with each one of the 119 primary hits at a 10 µmol/L concentration for 24 hours and the luciferase activity was then measured. Compounds passed the secondary screening if they were capable of inhibiting PAX3::FOXO1-responsive luciferase activity more than 70% without inhibiting the PGK-responsive luciferase signal. We identified six secondary hits as potential inhibitors of PAX3::FOXO1 (Fig. 2B; Supplementary Table S3). For further validation of PAX3::FOXO1 inhibitory activity, the six secondary hits were evaluated with a different PAX3::FOXO1-responsive promoter. The FP-RMS cell line RH30 and FN-RMS cell line RD were transfected with the PAX3::FOXO1-responsive *PDGFRA* luciferase reporter (40). Cells were treated with 10 µmol/L concentration of compounds for 48 hours and luciferase activity was measured (Fig. 2C). In this experiment, piperacetazine showed the most significant PAX3::FOXO1-specific response. Alcuronium choloride and didanosine did not inhibit the *PDGFRA* luciferase reporter. Carboplatin inhibition was less significant than inhibition by piperacetazine. Both NSC2805 and NSC697468 inhibited the *PDGFRA* luciferase reporter in FP-RMS cell line RH30, but they also inhibited the same reporter in FN-RMS cell line RD at a similar level and were therefore considered nonspecific. Therefore, piperacetazine was selected for further evaluation in additional functional assays (Fig. 2D). To calculate the binding affinity of piperacetazine to PAX3::FOXO1 protein, we performed SPR experiments using a Biacore T-200 instrument. Piperacetazine was injected at multiple different concentrations over the PAX3::FOXO1 protein bound to a CM5 chip surface. We detected an average binding affinity (K_D_) of 3.8 µmol/L (1.2 µmol/L S.E.) between the recombinant PAX3::FOXO1 protein and piperacetazine after six independent experiments. A representative binding sensorgram is presented in Fig. 2E.

To confirm that piperacetazine was capable of binding to endogenous PAX3::FOXO1 protein in RMS cells, we performed a cellular thermal shift assay (CETSA; refs. 41, 42). RH30 cell lysates were incubated with 30 µmol/L piperacetazine, to saturate binding sites, or DMSO for 30 minutes and then exposed to an increasing range of temperatures for 3 minutes. Proteins that aggregated because of heat were separated by centrifugation, and the proteins remaining in solution were then analyzed by Western blot analysis. The CETSA allowed us to determine that piperacetazine bound and stabilized endogenous PAX3::FOXO1, which remained in solution at higher temperatures (Fig. 2F). The experiment was repeated three times and band intensities for each temperature were quantified by densitometric analysis (Fig. 2G). The shift to the right was observed for PAX3::FOXO1 protein (top band in each blot, Fig. 2F) but not the wild-type FOXO1 (bottom band in each blot, Fig. 2F). This finding suggests that piperacetazine is capable of binding to endogenous PAX3::FOXO1 protein in the presence of its natural binding partners in RMS cells.

### Piperacetazine Inhibits the Transcriptional Activity of Endogenous PAX3::FOXO1 on Luciferase Reporters in Multiple Fusion-positive RMS Cells

**FIGURE 3:**
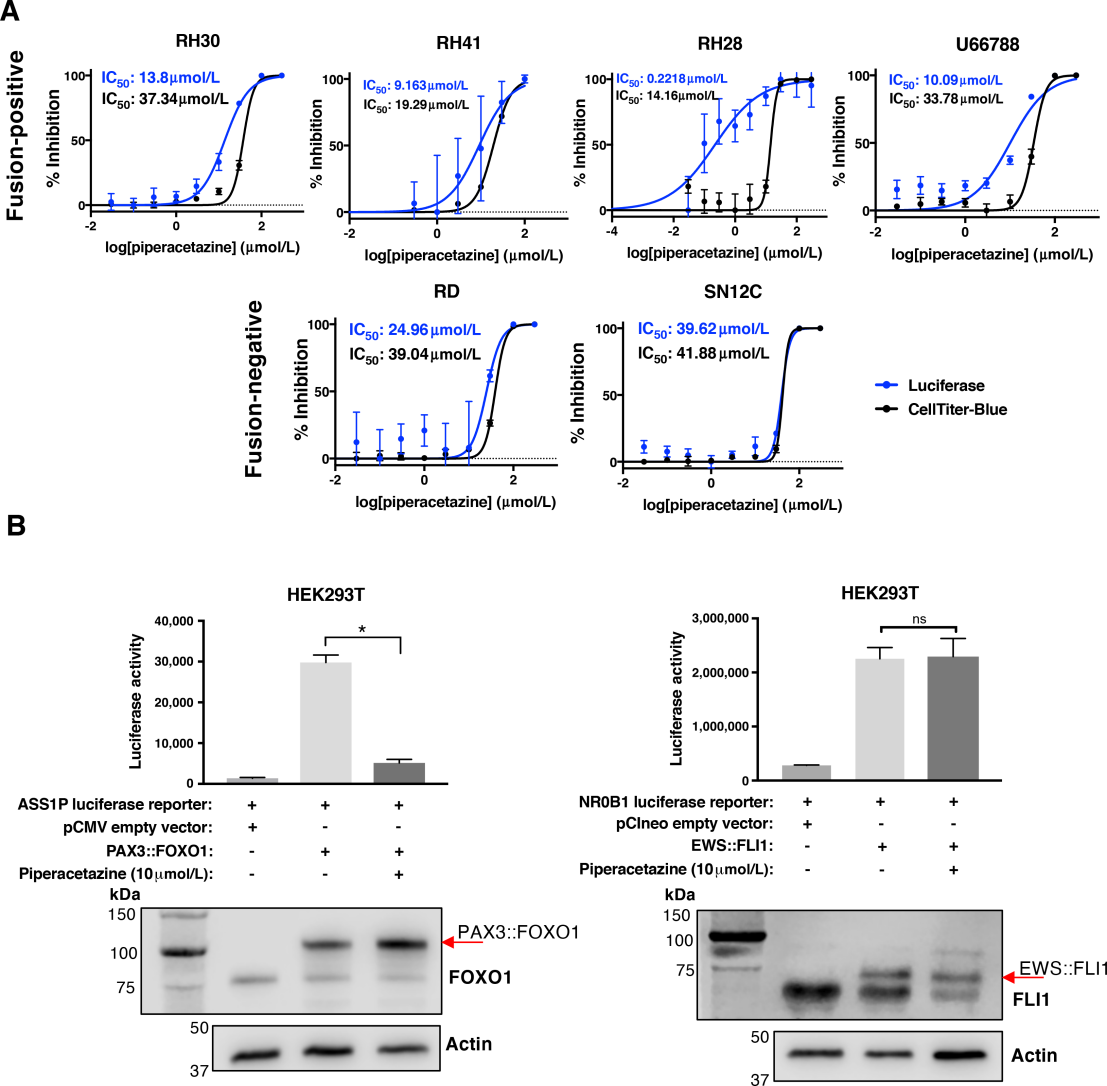
Piperacetazine inhibits endogenous PAX3::FOXO1 activity in multiple cell lines. **A,** The effect of piperacetazine treatment on cell viability was measured using the CellTiter-Blue assay (black lines). The effect of piperacetazine treatment on PAX3::FOXO1 activity was measured via luciferase assay (blue lines), using the PAX3::FOXO1-responsive pGL3-*PDGFRA* luciferase reporter (RH30, RH41, RH28, U66788, RD), and PGK reporter (SN12C, negative control). **B,** Left: Luciferase activity was measured in HEK293T cells transfected with PAX3::FOXO1-responsive *ASS1P* reporter and an empty or PAX3::FOXO1 expression vector. Cells were treated with DMSO or piperacetazine for 48 hours. Matching Western blots are shown under each bar. Right: The same experimental setup was repeated with EWS::FLI1-responsive *NR0B1* luciferase reporter and an EWS::FLI1 expression vector or empty vector. The cells were treated with the same concentration of piperacetazine or DMSO for 48 hours, and the normalized luciferase readouts and accompanying Western blots are shown. (*, *P* < 0.0001; Student *t* test; ns, *P* > 0.05)

To confirm that piperacetazine specifically inhibits endogenous PAX3::FOXO1’s transcriptional activity in FP-RMS cell lines, we again used the pGL3-PDGFRA reporter construct. This reporter construct was transfected into four FP-RMS cell lines, (RH30, RH28/L-PAM, RH41, U66788) and one FN-RMS (RD) cell line. We also included the non-RMS cell line SN12C with the PGK promoter luciferase construct as an additional negative control. The cells were treated with a range of piperacetazine concentrations, and after 48 hours, relative luciferase activity was measured by luminometer, while cell viability was measured by fluorescence using the CellTiter-Blue assay (Fig. 3A). In all four FP-RMS cell lines, luciferase activity was inhibited at a lower dose of piperacetazine than what was required to kill the cells, indicating a PAX3::FOXO1 specific activity. In contrast, in cells lacking PAX3::FOXO1, piperacetazine inhibited luciferase activity at doses that also induced cell death, indicating a nonspecific toxicity. This was observed as overlapping luciferase and cell viability curves. The consistent and reproducible shift of luciferase curves compared to cell viability curves in all four FP-RMS cell lines supports the hypothesis that piperacetazine inhibits transcriptional activity of PAX3::FOXO1 protein.

Further validation of piperacetazine as a specific PAX3::FOXO1 inhibitor occurred using a PAX3::FOXO1-responsive *ASS1P* luciferase reporter. An empty vector or *PAX3::FOXO1* construct was cotransfected with the *ASS1P* luciferase reporter into HEK293T cells. We evaluated the luciferase activity after 48 hours of DMSO or piperacetazine treatment (Fig. 3B, left). PAX3::FOXO1 induced a high luciferase signal that decreased significantly with piperacetazine treatment. We did not see this response to piperacetazine treatment when the HEK293T cells were transfected with a construct for a different fusion protein EWS::FLI1 and an EWS::FLI1-responsive *NR0B1* luciferase reporter as a negative control (Fig. 3B, right). These results further support the hypothesis that the transcriptional activity of PAX3::FOXO1 is selectively inhibited by piperacetazine. We also evaluated the inhibitory effect of piperacetazine on the PAX7::FOXO1 fusion protein in the same experimental system (Supplementary Fig. S2). Expression of PAX7::FOXO1 activated the *ASS1P* luciferase reporter in HEK293T cells, which was significantly reduced by 48 hours of piperacetazine treatment, demonstrating that piperacetazine may also inhibit the transcriptional activity of PAX7::FOXO1.

### Piperacetazine Inhibits Endogenous PAX3::FOXO1 Target Gene Expression

**FIGURE 4:**
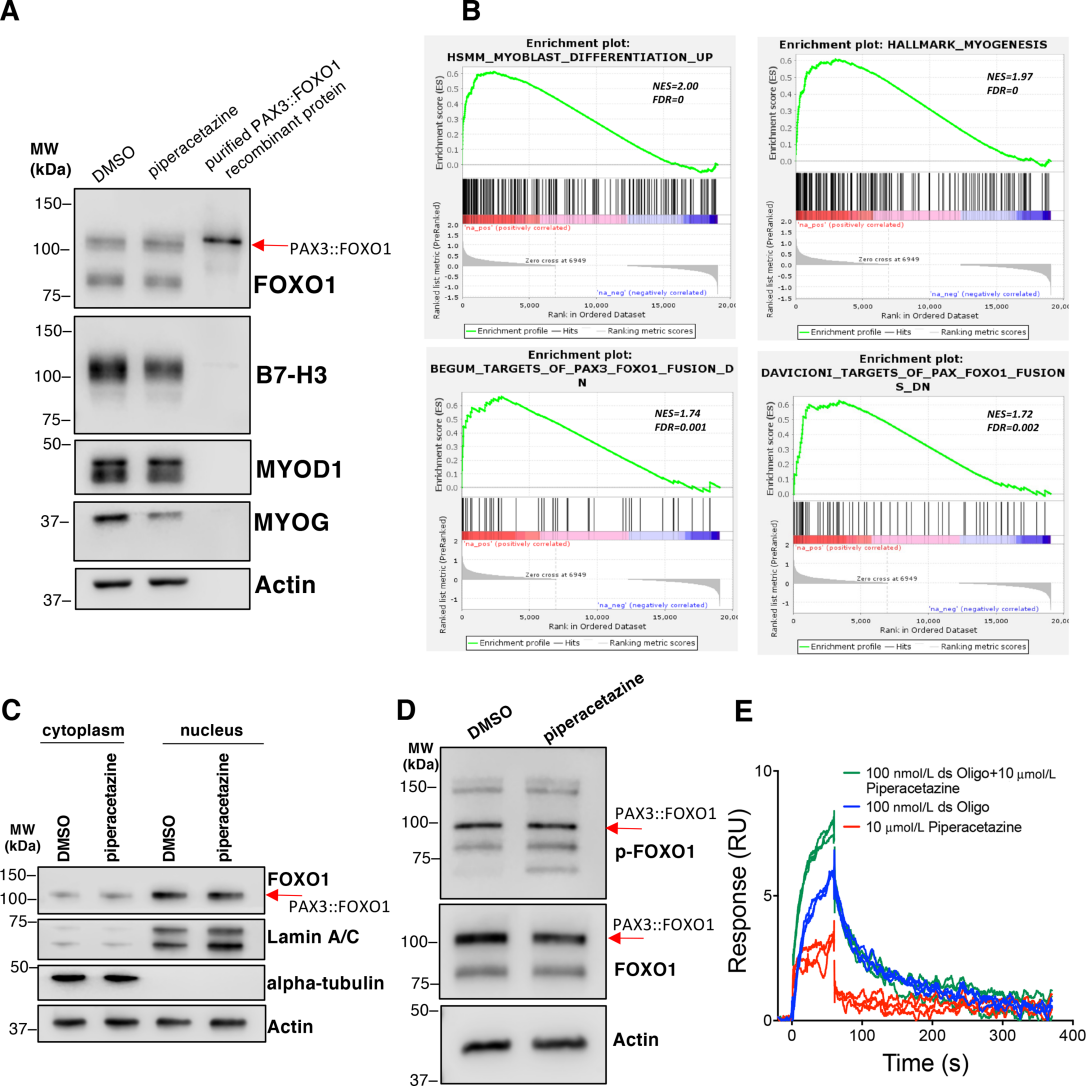
Piperacetazine alters the expression of PAX3::FOXO1 target genes. **A,** Protein expression of PAX3::FOXO1 target genes was evaluated by Western blot analysis in RH30 cells treated with 10 µmol/L piperacetazine for 6 days. **B,** RNA-seq was performed in fusion-positive RH30 cells treated with 30 µmol/L piperacetazine for 24 hours. The ranked gene expression list was compared with existing lists of genes using GSEA. Gene set descriptions: Top left: genes upregulated during human skeletal muscle myoblast differentiation. Top right: Hallmark genes during myogenesis. Bottom left: Genes downregulated in SAOS-2 (osteosarcoma) cells upon expression of PAX3::FOXO1. Bottom right: Genes downregulated in fusion-positive versus fusion-negative RMS cell lines. NES = normalized enrichment score, FDR = false discovery rate). **C,** Piperacetazine does not cause PAX3::FOXO1 to shift its intracellular localization. RH30 cells were treated with 10 µmol/L piperacetazine for 24 hours prior to cellular fractionation, which were analyzed via Western blot analysis. Lamin A/C and alpha-tubulin were used as positive controls for nuclear and cytoplasmic fractions, respectively. **D,** Piperacetazine does not alter PAX3::FOXO1 protein levels or phosphorylation of Ser256, as evaluated by Western blot analysis. RH30 cells were treated with 10 µmol/L piperacetazine or vehicle for 3 days. **E,** PAX3::FOXO1 protein was immobilized on a CM5 chip, and double-stranded PAX3::FOXO1 oligonucleotide (100 nmol/L), piperacetazine (10 µmol/L), or a combination of the two were injected over the chip surface. Piperacetazine did not inhibit DNA binding to PAX3::FOXO1.

To examine the ability of piperacetazine to inhibit transcriptional activity of PAX3::FOXO1 in FP-RMS cells, we chose to examine the expression of PAX3::FOXO1 target genes *MYOD1*, *MYOG*, and *B7-H3* (*CD276*) (24, 31, 43). RH30 cells were treated with a sublethal dose of piperacetazine for 6 days and the expression of target genes was evaluated by Western blot analysis (Fig. 4A). We observed reductions in the expression of all three proteins without any change in the levels of PAX3::FOXO1. In addition to reducing the expression of selected PAX3::FOXO1 target genes in RH30, we also investigated the changes in the global gene expression profile in response to piperacetazine in this cell line. We treated RH30 cells with 30 µmol/L piperacetazine or DMSO (negative control) for 24 hours, followed by RNA extraction and RNA-seq. We then used GSEA to characterize the biological effects of piperacetazine on RH30 cells. We observed a significant upregulation of skeletal muscle genes (HSMM_MYOBLAST_DIFFERENTIATION_UP, NES = 2.00, FDR = 0; HALLMARK_MYOGENESIS, NES = 1.97, FDR = 0) and genes known to be suppressed by PAX3::FOXO1 fusion protein (BEGUM_TARGETS_OF_PAX3_FOXO1_FUSION_DN, NES = 1.74, FDR = 0.001; DAVICIONI_TARGETS_OF_PAX_FOXO1_FUSIONS_DN, NES = 1.72, FDR = 0.002; Fig. 4B, Supplementary Table S4). These results suggest that the binding of piperacetazine to PAX3::FOXO1 specifically changes the expression profiles of the downstream fusion target genes with a strong antagonistic effect against the fusion gene functions. Interestingly, we also observed a correlation with some gene sets suggesting a PAX3::FOXO1 agonistic profile too (Supplementary Table S4).

We investigated potential molecular mechanisms for piperacetazine-mediated inhibition of PAX3::FOXO1 activity. We first evaluated whether piperacetazine could be inhibiting expression of endogenous PAX3::FOXO1 protein or accelerating its degradation, either of which would result in reduced PAX3::FOXO1 protein levels. We did not observe a significant change in PAX3::FOXO1 protein levels following piperacetazine treatment (Fig. 4A). Another possibility that was considered was that piperacetazine could be altering intracellular localization of PAX3::FOXO1 protein. Preventing PAX3::FOXO1 entry to the nucleus or facilitating its export to the cytoplasm would prevent PAX3::FOXO1 from performing its transcriptional activity. When we investigated protein levels in subcellular fractions, we did not observe any shift in nuclear or cytoplasmic localization of PAX3::FOXO1 with piperacetazine treatment (Fig. 4C). It is also possible to regulate PAX3::FOXO1 activity by modulating its phosphorylation, but we did not observe any change in the levels of PAX3::FOXO1 phosphorylation (Fig. 4D). The antibody used in this experiment was specific only for phosphorylation at Ser256, so it is possible that other phosphorylation sites may be altered. Finally, it was also possible that piperacetazine could prevent PAX3::FOXO1 binding to DNA to block its transcriptional activity. However, when we evaluated the DNA-binding ability of purified PAX3::FOXO1 protein by SPR, we did not observe any inhibition by piperacetazine. Even at 100-fold molar excess, piperacetazine did not inhibit DNA oligonucleotide binding to recombinant PAX3::FOXO1 protein (Fig. 4E). This result suggests that PAX3::FOXO1 can simultaneously bind to both DNA and piperacetazine. Therefore, we concluded that piperacetazine does not block PAX3::FOXO1 from binding to DNA as a primary mechanism of action. Instead, we concluded that the most likely mechanism of action for piperacetazine was the prevention of protein–protein interactions involving PAX3::FOXO1. For this reason, we also concluded that the correlation with different GSEA phenotypes might be dependent on which specific protein-protein interactions were disrupted by piperacetazine in different cell lines.

### Piperacetazine Inhibits Anchorage-independent FP-RMS Growth in Soft Agar but not in Two-dimensional Culture

**FIGURE 5:**
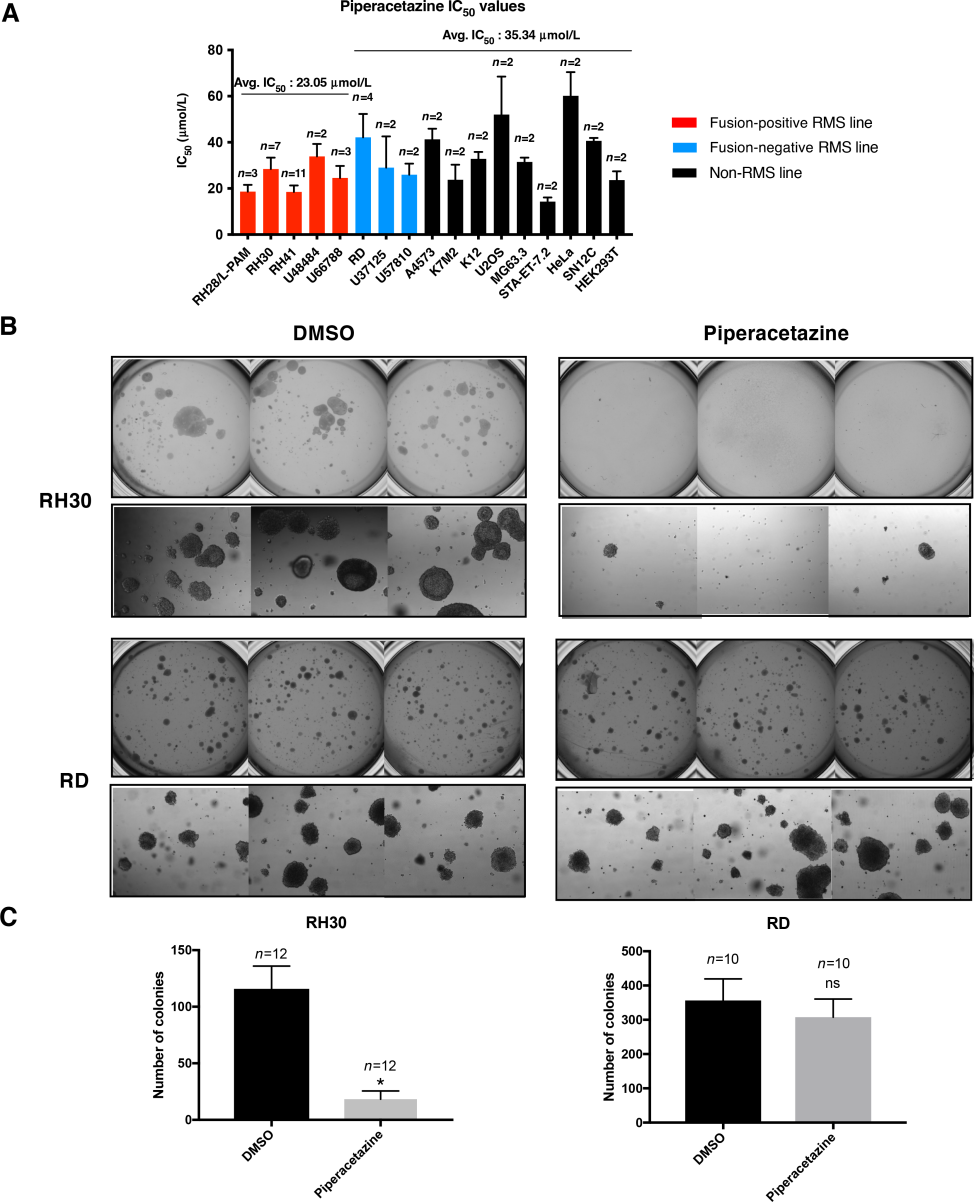
Piperacetazine inhibits anchorage-independent growth of FP-RMS cells. **A,** Average 48 hour IC_50_ values for indicated cell lines. Sample size indicated above each bar. Red bars: FP-RMS, blue bars: FN-RMS, black bars: non-RMS. U48484, U66788, U37125, and U57810 are cell lines from transgenic RMS mouse models. **B,** RH30 and RD colonies in soft agar with DMSO or 15 µmol/L piperacetazine treatment. The top row images are whole well images taken with Gelcount and the bottom row images are microscope images of the colonies at 40X magnification. **C,** Gelcount quantification of colonies in B (*, *P* < 0.0001; Student *t* test; ns, *P* > 0.05).

It has been reported that FP-RMS cells may be dependent on the PAX3::FOXO1 fusion for survival (44–46). However, there were other studies suggesting that FP-RMS cells can acquire the ability to survive in the absence of PAX3::FOXO1 (17). We compared piperacetazine's effects on cell viability for five FP-RMS cell lines, three FN-RMS cell lines, and nine non-RMS cell lines using a CellTiter-Blue assay in cell culture. Cells were treated with a range of piperacetazine concentrations, and cell viability was measured at 48 hours. The results showed that IC_50_ values in FP-RMS cell lines were on average slightly lower (42% difference, *P* value of 0.002 by Student *t* test) than those in FN-RMS and non-RMS cell lines (Fig. 5A; Supplementary Fig. S3). This observation was consistent with our experience in FP-RMS cells when PAX3::FOXO1 expression was inhibited with siRNA. Both RH30 and U66788 cells showed comparable proliferation rates following a significant reduction of PAX3::FOXO1 protein expression by fusion-specific siRNA (Supplementary Fig. S4A and S4B). Therefore, we concluded that cell viability was not a reliable readout for measuring PAX3::FOXO1 activity in cultured FP-RMS cell lines on plastic surfaces. We evaluated anchorage-independent growth of RH30 cells with knockdown of PAX3::FOXO1 in a soft agar colony formation assay. Reduction in PAX3::FOXO1 protein expression inhibited anchorage-independent growth, suggesting that three-dimensional colony formation in soft agar is a more reliable readout for measuring PAX3::FOXO1 activity in RH30 cells. This experiment was performed twice, and representative images are provided in Supplementary Fig. S4C.

We then evaluated the effect of piperacetazine on anchorage-independent growth of a FP-RMS cell line RH30 and FN-RMS cell line RD. Both cells formed measurable colonies in soft agar in 14–21 days. When they were treated with 15 µmol/L piperacetazine in the top agar layer, we observed a significant decrease in colony formation only in FP-RMS cell line RH30 but not in FN-RMS cell line RD. The soft agar experiment was performed twice. Representative images are shown in Fig. 5B and quantification of the entire plates is provided in Fig. 5C. The selective inhibitory effect of piperacetazine on RH30 compared with RD further supported the hypothesis that piperacetazine selectively targets PAX3::FOXO1 in RMS cells.

To evaluate the full antineoplastic activity of piperacetazine, we performed a xenograft study by injecting RH30 cells to the gastrocnemius muscle of immunocompromised mice. When the animals developed palpable tumors, they were randomized to control (DMSO) or treatment groups (20 mg/kg/day piperacetazine). An initial dose of 30 mg/kg was given on day 1, and animals exhibited labored breathing, markedly reduced sensitivity to handling, and sedation for 4 hours or more. Because of concerns about access to food and water, the dose was lowered to 20 mg/kg/day to reduce the possibility of dehydration or weight loss. Piperacetazine treatment slowed the tumor growth rate but the difference in tumor volume between two groups was not statistically significant (*P* = 0.0683; Supplementary Fig. S5A). We also tested the antineoplastic activity of piperacetazine in a second mouse model where PAX3::FOXO1 is expressed under a doxycycline-regulated promoter in an immortalized human Duchenne muscular dystrophy myoblast cell line (Dbt) expressing MYCN (17). Cells were implanted in the gastrocnemius muscle of NOD-SCID mice. Animals were fed doxycycline-containing (625 mg/kg) diet beginning 3 days prior to cell line injection. When the tumors were palpable, the animals were randomized to three groups; (i) a positive control group for which the diet was switched to regular pellets without doxycycline, and received placebo injections (DMSO); (ii) a negative control group that was fed a doxycycline-containing diet and received placebo injections (DMSO); and (iii) an experimental group where animals were also kept on doxycycline-containing diet and received intraperitoneal piperacetazine injections for 2 weeks (20 mg/kg/day). As expected, with the removal of doxycycline from the diet and loss of PAX3::FOXO1 expression, the tumors in the positive control group stopped growing. Those in the negative control group rapidly grew and reached the size limit for euthanasia in 2 weeks. Piperacetazine treatment did not have any effect on the tumor growth in this model (Supplementary Fig. S5B).

Although piperacetazine did not demonstrate the ability to prevent PAX3::FOXO1-mediated tumorigenesis in these two mouse models, we hypothesized that it might act in synergy with existing chemotherapeutic agents. Previous research has shown that entinostat, a histone deacetylase inhibitor with the ability to prevent PAX3::FOXO1 transcription, is able to act in synergy with vincristine to prevent tumorigenesis in specific mouse models of ARMS (47). We tested piperacetazine in combination with four chemotherapeutic agents *in vitro* and analyzed the results using SynergyFinder software. Piperacetazine exhibited synergy with JQ-1, vincristine, and entinostat, although this synergy was present in both FP-RMS and FN-RMS cells (Supplementary Fig. S6). Therefore, it is worth considering future *in vivo* xenograft studies with piperacetazine in combination with JQ-1, vincristine, or entinostat. We consider piperacetazine as a lead molecule or a scaffold for the design of more potent PAX3::FOXO1 inhibitors using a structure activity relationship (SAR) technique or as part of a combination therapy, but not as a treatment option in and of itself as a single agent.

## Discussion

PAX3::FOXO1 is a key driver of oncogenesis as a defining protein of FP-RMS. Some compounds capable of inhibiting downstream targets of PAX3::FOXO1 or phenocopying the gene expression profile caused by the loss of PAX3::FOXO1 are reported, yet these compounds have not become useful for patients yet. Our approach directly targeted the PAX3::FOXO1 protein itself. To do this, we used SPR to screen 3,894 small molecules for their binding capability. We tested primary SPR hits in a functional assay and identified six lead compounds. These six compounds were evaluated using a variety of functional assays, and piperacetazine was selected on the basis of its ability to specifically inhibit PAX3::FOXO1-driven reporters and anchorage-independent growth of FP-RMS cells.

We identified piperacetazine as a small molecule directly binding to PAX3::FOXO1 with low µmol/L affinity and inhibiting its activity. We did not observe a change in PAX3::FOXO1 protein expression level, localization, phosphorylation, or DNA binding in response to treatment with piperacetazine. These findings suggest that piperacetazine's effects on PAX3::FOXO1 protein activity may be due in part to a disruption of protein–protein interactions. We had a similar experience when we discovered small-molecule inhibitors of EWS::FLI1 protein in Ewing sarcoma. YK-4-279 was able to directly bind to EWS::FLI1 protein and inhibit its oncogenic properties (48). Similar to piperacetazine, YK-4-279 was not preventing EWS::FLI1 binding to DNA. We discovered that the primary mechanism of action for YK-4-279 was through inhibiting specific protein–protein interactions involving EWS::FLI1, which included an RNA helicase (DHX9) and components of the splicing machinery (49, 50). A similar approach is required to identify which specific protein–protein interactions involving PAX3::FOXO1 are inhibited by piperacetazine in FP-RMS cells in future studies.

It is possible that piperacetazine may prevent only some PAX3::FOXO1 protein–protein interactions, and therefore may inhibit only a subset of PAX3::FOXO1 functions. The overall gene expression profile associated with PAX3::FOXO1 activity is likely to be context dependent and may be dependent on the cell line, culture conditions and the presence or absence of key protein partners. Therefore, it is plausible to expect that piperacetazine may inhibit some protein–protein interactions involving PAX3::FOXO1 but not others. Hence the piperacetazine-mediated gene expression profile may only partially match the complete loss of the PAX3::FOXO1 protein. We plan to conduct future experiments to determine which specific protein–protein interactions may be disrupted by piperacetazine treatment.

We did not observe a strong antitumor activity of piperacetazine in RMS xenograft experiments. There are two potential explanations for the lack of antitumor activity in these *in vivo* experiments. First, the pharmacology of piperacetazine in mice may have led to low drug levels in the tumor tissue. Because of the lethargic effects of piperacetazine on mice, we were not able to administer higher doses. Future experiments with a proper pharmacokinetics and pharmacodynamics analysis can rule out this possibility. Second, is that even though piperacetazine can inhibit the transcriptional activity of PAX3::FOXO1 *in vivo*, this may not be enough to provide an antitumor phenotype. The latter possibility suggests that PAX3::FOXO1 may have additional properties that are important for its oncogenic functions.

Because of its relatively weak binding affinity and lack of *in vivo* efficacy, piperacetazine alone should not be considered an ultimate inhibitor of PAX3::FOXO1 that can be used in the clinic at this time. However, piperacetazine can still serve as a useful research tool to study the molecular mechanism of PAX3::FOXO1 in experimental models. More importantly, through a SAR campaign, piperacetazine can be used as a scaffold to design and synthesize novel compounds that may have much stronger binding affinity, better solubility and improved *in vivo* efficacy. A successful SAR approach is likely to improve pharmacokinetic and pharmacodynamic properties and achieve better therapeutic outcomes (51).

We selected piperacetazine for its ability to inhibit PAX3::FOXO1 activity in multiple functional assays. Other potential PAX3::FOXO1 inhibitors identified by our screening method (Fig. 2B; Supplementary Table S3) may be binding to different regions of PAX3::FOXO1 and may be capable of inhibiting different functions of PAX3::FOXO1 through preventing alternative protein–protein interactions. It may be worth considering a future synergy experiment to evaluate all PAX3::FOXO1 binders with each other. In addition, any compound identified as a primary hit by our initial screen (Supplementary Table S2), may potentially be a good candidate for a proteolysis targeting chimera (PROTAC) approach. PROTACs can significantly reduce the half-life of PAX3::FOXO1 protein by recruiting an E3 ligase, which then ubiquitinates the protein, targeting it for degradation (52).

Our primary goal was to identify small molecules that can directly bind and inhibit PAX3::FOXO1 protein. However, in the secondary screening experiment, there were several compounds that appeared to increase the transcriptional activity of PAX3::FOXO1 in the luciferase assay (Fig. 2B). The top 10 compounds that showed more than 5-fold activation of PAX3::FOXO1-responsive reporter assay without affecting the negative control are listed in Supplementary Table S5. If validated, these compounds could be useful research tools to further investigate the functions of PAX3::FOXO1 in FP-RMS cells.

In summary, our data demonstrated that piperacetazine binds directly to PAX3::FOXO1 in FP-RMS cells and inhibits its transcriptional activity, which results in reduced growth in soft agar. Piperacetazine may be used as a laboratory research tool to study PAX3::FOXO1 function and can be improved by medicinal chemistry to better molecules with enhanced antitumor activity for clinical applications.

## Supplementary Material

Supplementary Tables 1, 2, and 3 Supplementary Tables 1, 2, and 3

Supplementary Table 4 Supplementary Table 4

Supplementary Table 5 Supplementary Table 5

Supplementary Figure 1 Nucleotide and amino acid sequences of PAX3::FOXO1 gene in bacterial expression plasmid pET104.1 are provided.

Supplementary Figure 2 Luciferase assay performed to measure the effect of piperacetazine on PAX7::FOXO1 activity.

Supplementary Figure 3 Piperacetazine IC50 values across various cell lines.

Supplementary Figure 4 Reduction of PAX3::FOXO1 protein expression does not alter proliferation rates of FP-RMS cell lines but reduces anchorage-independent growth.

Supplementary Figure 5 Piperacetazine is not effective in preventing PAX3::FOXO1- mediated tumorigenesis at the tested dose.

Supplementary Figure 6 Synergy between piperacetazine and other chemotherapeutic drugs are similar between the RH30 and RD cell lines.

## References

[1] Ries LAG , SEER Program. Cancer incidence and survival among children and adolescents: United States SEER program 1975–1995 / [edited by Lynn A. Gloecker Ries … et al.]. Bethesda, MD: National Cancer Institute; 1999. vi, p. 114–21.

[2] Rudzinski ER , Teot LA, Anderson JR, Moore J, Bridge JA, Barr FG, . Dense pattern of embryonal rhabdomyosarcoma, a lesion easily confused with alveolar rhabdomyosarcoma: a report from the Soft Tissue Sarcoma Committee of the Children's Oncology Group. Am J Clin Pathol 2013;140:82–90. 23765537 10.1309/AJCPA1WN7ARPCMKQ PMC4624292

[3] McEvoy MT , Siegel DA, Dai S, Okcu MF, Zobeck M, Venkatramani R, . Pediatric rhabdomyosarcoma incidence and survival in the United States: an assessment of 5656 cases, 2001–17. Cancer Med 2022;12:3644–56. 36069287 10.1002/cam4.5211 PMC9939205

[4] Sebire NJ , Malone M. Myogenin and MyoD1 expression in paediatric rhabdomyosarcomas. J Clin Pathol 2003;56:412–6. 12783965 10.1136/jcp.56.6.412 PMC1769965

[5] Kumar S , Perlman E, Harris CA, Raffeld M, Tsokos M. Myogenin is a specific marker for rhabdomyosarcoma: an immunohistochemical study in paraffin-embedded tissues. Mod Pathol 2000;13:988–93. 11007039 10.1038/modpathol.3880179

[6] Kahn HJ , Yeger H, Kassim O, Jorgensen AO, MacLennan DH, Baumal R, . Immunohistochemical and electron microscopic assessment of childhood rhabdomyosarcoma. Increased frequency of diagnosis over routine histologic methods. Cancer 1983;51:1897–903. 6131739 10.1002/1097-0142(19830515)51:10<1897::aid-cncr2820511023>3.0.co;2-7

[7] Dias P , Kumar P, Marsden HB, Morris-Jones PH, Birch J, Swindell R, . Evaluation of desmin as a diagnostic and prognostic marker of childhood rhabdomyosarcomas and embryonal sarcomas. Br J Cancer 1987;56:361–5. 3311112 10.1038/bjc.1987.203 PMC2002207

[8] de Jong AS , van Kessel-van Vark M, Albus-Lutter CE, van Raamsdonk W, Voute PA. Skeletal muscle actin as tumor marker in the diagnosis of rhabdomyosarcoma in childhood. Am J Surg Pathol 1985;9:467–74. 3911779 10.1097/00000478-198507000-00001

[9] Skapek SX , Ferrari A, Gupta AA, Lupo PJ, Butler E, Shipley J, . Rhabdomyosarcoma. Nat Rev Dis Primers 2019;5:1. 30617281 10.1038/s41572-018-0051-2 PMC7456566

[10] Mascarenhas L , Chi YY, Hingorani P, Anderson JR, Lyden ER, Rodeberg DA, . Randomized phase II trial of bevacizumab or temsirolimus in combination with chemotherapy for first relapse rhabdomyosarcoma: a report from the Children's Oncology Group. J Clin Oncol 2019;37:2866–74. 31513481 10.1200/JCO.19.00576 PMC6823886

[11] Barr FG , Galili N, Holick J, Biegel JA, Rovera G, Emanuel BS. Rearrangement of the PAX3 paired box gene in the paediatric solid tumour alveolar rhabdomyosarcoma. Nat Genet 1993;3:113–7. 8098985 10.1038/ng0293-113

[12] Davis RJ , D'Cruz CM, Lovell MA, Biegel JA, Barr FG. Fusion of PAX7 to FKHR by the variant t(1;13)(p36;q14) translocation in alveolar rhabdomyosarcoma. Cancer Res 1994;54:2869–72. 8187070

[13] Skapek SX , Anderson J, Barr FG, Bridge JA, Gastier-Foster JM, Parham DM, . PAX-FOXO1 fusion status drives unfavorable outcome for children with rhabdomyosarcoma: a Children's Oncology Group report. Pediatr Blood Cancer 2013;60:1411–7. 23526739 10.1002/pbc.24532 PMC4646073

[14] Fredericks WJ , Galili N, Mukhopadhyay S, Rovera G, Bennicelli J, Barr FG, . The PAX3-FKHR fusion protein created by the t(2;13) translocation in alveolar rhabdomyosarcomas is a more potent transcriptional activator than PAX3. Mol Cell Biol 1995;15:1522–35. 7862145 10.1128/mcb.15.3.1522 PMC230376

[15] Bennicelli JL , Edwards RH, Barr FG. Mechanism for transcriptional gain of function resulting from chromosomal translocation in alveolar rhabdomyosarcoma. Proc Natl Acad Sci U S A 1996;93:5455–9. 8643596 10.1073/pnas.93.11.5455 PMC39267

[16] Bennicelli JL , Advani S, Schafer BW, Barr FG. PAX3 and PAX7 exhibit conserved cis-acting transcription repression domains and utilize a common gain of function mechanism in alveolar rhabdomyosarcoma. Oncogene 1999;18:4348–56. 10439042 10.1038/sj.onc.1202812

[17] Pandey PR , Chatterjee B, Olanich ME, Khan J, Miettinen MM, Hewitt SM, . PAX3-FOXO1 is essential for tumour initiation and maintenance but not recurrence in a human myoblast model of rhabdomyosarcoma. J Pathol 2017;241:626–37. 28138962 10.1002/path.4867 PMC5357165

[18] Kikuchi K , Hettmer S, Aslam MI, Michalek JE, Laub W, Wilky BA, . Cell-cycle dependent expression of a translocation-mediated fusion oncogene mediates checkpoint adaptation in rhabdomyosarcoma. PLoS Genet 2014;10:e1004107. 24453992 10.1371/journal.pgen.1004107 PMC3894165

[19] Raze T , Lapouble E, Lacour B, Guissou S, Defachelles AS, Gaspar N, . PAX-FOXO1 fusion status in children and adolescents with alveolar rhabdomyosarcoma: impact on clinical, pathological, and survival features. Pediatr Blood Cancer 2023;70:e30228. 36722003 10.1002/pbc.30228

[20] Sorensen PH , Lynch JC, Qualman SJ, Tirabosco R, Lim JF, Maurer HM, . PAX3-FKHR and PAX7-FKHR gene fusions are prognostic indicators in alveolar rhabdomyosarcoma: a report from the children's oncology group. J Clin Oncol 2002;20:2672–9. 12039929 10.1200/JCO.2002.03.137

[21] Jothi M , Mal M, Keller C, Mal AK. Small molecule inhibition of PAX3-FOXO1 through AKT activation suppresses malignant phenotypes of alveolar rhabdomyosarcoma. Mol Cancer Ther 2013;12:2663–74. 24107448 10.1158/1535-7163.MCT-13-0277 PMC3858449

[22] Abraham J , Nunez-Alvarez Y, Hettmer S, Carrio E, Chen HI, Nishijo K, . Lineage of origin in rhabdomyosarcoma informs pharmacological response. Genes Dev 2014;28:1578–91. 25030697 10.1101/gad.238733.114 PMC4102765

[23] Herrero Martin D , Boro A, Schafer BW. Cell-based small-molecule compound screen identifies fenretinide as potential therapeutic for translocation-positive rhabdomyosarcoma. PLoS One 2013;8:e55072. 23372815 10.1371/journal.pone.0055072 PMC3555977

[24] Gryder BE , Yohe ME, Chou HC, Zhang X, Marques J, Wachtel M, . PAX3-FOXO1 establishes myogenic super enhancers and confers BET bromodomain vulnerability. Cancer Discov 2017;7:884–99. 28446439 10.1158/2159-8290.CD-16-1297 PMC7802885

[25] Ghayad SE , Rammal G, Sarkis O, Basma H, Ghamloush F, Fahs A, . The histone deacetylase inhibitor Suberoylanilide Hydroxamic Acid (SAHA) as a therapeutic agent in rhabdomyosarcoma. Cancer Biol Ther 2019;20:272–83. 30307360 10.1080/15384047.2018.1529093 PMC6370390

[26] Bharathy N , Svalina MN, Settelmeyer TP, Cleary MM, Berlow NE, Airhart SD, . Preclinical testing of the glycogen synthase kinase-3beta inhibitor tideglusib for rhabdomyosarcoma. Oncotarget 2017;8:62976–83. 28968964 10.18632/oncotarget.18520 PMC5609896

[27] Kaylani SZ , Xu J, Srivastava RK, Kopelovich L, Pressey JG, Athar M. Rapamycin targeting mTOR and hedgehog signaling pathways blocks human rhabdomyosarcoma growth in xenograft murine model. Biochem Biophys Res Commun 2013;435:557–61. 23665330 10.1016/j.bbrc.2013.05.001

[28] Li SQ , Cheuk AT, Shern JF, Song YK, Hurd L, Liao H, . Targeting wild-type and mutationally activated FGFR4 in rhabdomyosarcoma with the inhibitor ponatinib (AP24534). PLoS One 2013;8:e76551. 24124571 10.1371/journal.pone.0076551 PMC3790700

[29] Haworth K , Jones LM, Mandel W. Clinical experience with a new phenothiazine (piperacetazine). Am J Psychiatry 1961;117:749–50. 13712423 10.1176/ajp.117.8.749

[30] Li P , Snyder GL, Vanover KE. Dopamine targeting drugs for the treatment of schizophrenia: past, present and future. Curr Top Med Chem 2016;16:3385–403. 27291902 10.2174/1568026616666160608084834 PMC5112764

[31] Ahn EH , Mercado GE, Lae M, Ladanyi M. Identification of target genes of PAX3-FOXO1 in alveolar rhabdomyosarcoma. Oncol Rep 2013;30:968–78. 23733015 10.3892/or.2013.2513 PMC3776721

[32] Bharathy N , Cleary MM, Kim JA, Nagamori K, Crawford KA, Wang E, . SMARCA4 biology in alveolar rhabdomyosarcoma. Oncogene 2022;41:1647–56. 35094009 10.1038/s41388-022-02205-0 PMC9985831

[33] Aslam MI , Abraham J, Mansoor A, Druker BJ, Tyner JW, Keller C. PDGFRbeta reverses EphB4 signaling in alveolar rhabdomyosarcoma. Proc Natl Acad Sci U S A 2014;111:6383–8. 24733895 10.1073/pnas.1403608111 PMC4035936

[34] Cleary MM , Mansoor A, Settelmeyer T, Ijiri Y, Ladner KJ, Svalina MN, . NFκB signaling in alveolar rhabdomyosarcoma. Dis Model Mech 2017;10:1109–15. 28883017 10.1242/dmm.030882 PMC5611971

[35] Love MI , Huber W, Anders S. Moderated estimation of fold change and dispersion for RNA-seq data with DESeq2. Genome Biol 2014;15:550. 25516281 10.1186/s13059-014-0550-8 PMC4302049

[36] Subramanian A , Tamayo P, Mootha VK, Mukherjee S, Ebert BL, Gillette MA, . Gene set enrichment analysis: a knowledge-based approach for interpreting genome-wide expression profiles. Proc Natl Acad Sci U S A 2005;102:15545–50. 16199517 10.1073/pnas.0506580102 PMC1239896

[37] Zheng S , Wang W, Aldahdooh J, Malyutina A, Shadbahr T, Tanoli Z, . SynergyFinder plus: toward better interpretation and annotation of drug combination screening datasets. Genomics Proteomics Bioinformatics 2022;20:587–96. 35085776 10.1016/j.gpb.2022.01.004 PMC9801064

[38] Duan F , Simeone S, Wu R, Grady J, Mandoiu I, Srivastava PK. Area under the curve as a tool to measure kinetics of tumor growth in experimental animals. J Immunol Methods 2012;382:224–8. 22698786 10.1016/j.jim.2012.06.005

[39] Jothi M , Nishijo K, Keller C, Mal AK. AKT and PAX3-FKHR cooperation enforces myogenic differentiation blockade in alveolar rhabdomyosarcoma cell. Cell Cycle 2012;11:895–908. 22333587 10.4161/cc.11.5.19346 PMC3323795

[40] Taniguchi E , Nishijo K, McCleish AT, Michalek JE, Grayson MH, Infante AJ, . PDGFR-A is a therapeutic target in alveolar rhabdomyosarcoma. Oncogene 2008;27:6550–60. 18679424 10.1038/onc.2008.255 PMC2813858

[41] Martinez Molina D , Jafari R, Ignatushchenko M, Seki T, Larsson EA, Dan C, . Monitoring drug target engagement in cells and tissues using the cellular thermal shift assay. Science 2013;341:84–7. 23828940 10.1126/science.1233606

[42] Jafari R , Almqvist H, Axelsson H, Ignatushchenko M, Lundback T, Nordlund P, . The cellular thermal shift assay for evaluating drug target interactions in cells. Nat Protoc 2014;9:2100–22. 25101824 10.1038/nprot.2014.138

[43] Kanayama T , Miyachi M, Sugimoto Y, Yagyu S, Kikuchi K, Tsuchiya K, . Reduced B7-H3 expression by PAX3-FOXO1 knockdown inhibits cellular motility and promotes myogenic differentiation in alveolar rhabdomyosarcoma. Sci Rep 2021;11:18802. 34552155 10.1038/s41598-021-98322-z PMC8458399

[44] Bernasconi M , Remppis A, Fredericks WJ, Rauscher FJ 3rd, Schafer BW. Induction of apoptosis in rhabdomyosarcoma cells through down-regulation of PAX proteins. Proc Natl Acad Sci U S A 1996;93:13164–9. 8917562 10.1073/pnas.93.23.13164 PMC24064

[45] Ebauer M , Wachtel M, Niggli FK, Schafer BW. Comparative expression profiling identifies an *in vivo* target gene signature with TFAP2B as a mediator of the survival function of PAX3/FKHR. Oncogene 2007;26:7267–81. 17525748 10.1038/sj.onc.1210525

[46] Kikuchi K , Tsuchiya K, Otabe O, Gotoh T, Tamura S, Katsumi Y, . Effects of PAX3-FKHR on malignant phenotypes in alveolar rhabdomyosarcoma. Biochem Biophys Res Commun 2008;365:568–74. 18022385 10.1016/j.bbrc.2007.11.017

[47] Bharathy N , Berlow NE, Wang E, Abraham J, Settelmeyer TP, Hooper JE, . The HDAC3-SMARCA4-miR-27a axis promotes expression of the PAX3:FOXO1 fusion oncogene in rhabdomyosarcoma. Sci Signal 2018;11:eaau7632. 30459282 10.1126/scisignal.aau7632 PMC6432638

[48] Erkizan HV , Kong Y, Merchant M, Schlottmann S, Barber-Rotenberg JS, Yuan L, . A small molecule blocking oncogenic protein EWS-FLI1 interaction with RNA helicase A inhibits growth of Ewing's sarcoma. Nat Med 2009;15:750–6. 19584866 10.1038/nm.1983 PMC2777681

[49] Erkizan HV , Schneider JA, Sajwan K, Graham GT, Griffin B, Chasovskikh S, . RNA helicase A activity is inhibited by oncogenic transcription factor EWS-FLI1. Nucleic Acids Res 2015;43:1069–80. 25564528 10.1093/nar/gku1328 PMC4333382

[50] Selvanathan SP , Graham GT, Erkizan HV, Dirksen U, Natarajan TG, Dakic A, . Oncogenic fusion protein EWS-FLI1 is a network hub that regulates alternative splicing. Proc Natl Acad Sci U S A 2015;112:E1307–16. 25737553 10.1073/pnas.1500536112 PMC4371969

[51] Zhang Z , Tang W. Drug metabolism in drug discovery and development. Acta Pharm Sin B 2018;8:721–32. 30245961 10.1016/j.apsb.2018.04.003 PMC6146880

[52] Sakamoto KM , Kim KB, Kumagai A, Mercurio F, Crews CM, Deshaies RJ. Protacs: chimeric molecules that target proteins to the Skp1-Cullin-F box complex for ubiquitination and degradation. Proc Natl Acad Sci U S A 2001;98:8554–9. 11438690 10.1073/pnas.141230798 PMC37474

