# Supplementary material for: Piperacetazine Directly Binds to the PAX3::FOXO1 Fusion Protein and Inhibits Its Transcriptional Activity
Source: Cancer Res Commun. 2023 Oct 6;3(10):2030–43. doi: 10.1158/2767-9764.CRC-23-0119 (PMC10557868; doi:10.1158/2767-9764.CRC-23-0119)
Supplement: Supplementary Tables 1, 2, and 3 [file crc-23-0119-s01.docx]

|  | **Sample Name** | **D5S818** | **D13S317** | **D7S820** | **D16S539** | **VWA** | **TH01** | **AM** | **TPOX** | **CSF1PO** | **D3S1358** | **D21S11** | **D18S51** | **Penta_E** | **Penta_D** | **D8S1179** | **FGA** | **Multiple Profiles Seen** |  |  |  |
| --- | --- | --- | --- | --- | --- | --- | --- | --- | --- | --- | --- | --- | --- | --- | --- | --- | --- | --- | --- | --- | --- |
| **Human cell lines** | **MG63.3** | 11 12 | 11 11 | 10 10 | 11 11 | 16 19 | 9.3 9.3 | X X | 8 11 | 10 12 | 15 15 | 30 30 | 16 16 | 11 12 | 9 13 | 13 13 | 21 25 | NO |  |  |  |
|  |  |  |  |  |  |  |  |  |  |  |  |  |  |  |  |  |  |  |  |  |  |
|  | **RD** | 11 |  | 8 | 10 | 18 | 9.3 | X X | 9 | 10 | 15 | 28 | 18 | 12 | 11 | 11 | 20 21 | NO |  |  |  |
|  |  | 11 |  | 12 | 10 | 18 | 9.3 |  | 9 | 11 | 17 | 28 | 18 | 12 | 11 | 15 |  |  |  |  |  |
|  | **RH28/L-PAM** | 11 11 | 10 10 | 8 12 | 8 9 | 16 18 | 9 9.3 | X Y | 8 8 | 11 11 | 15 16 | 27 28 30 | 14 18 | 7 | 10 12 | 11 15 | 21 23 | NO |  |  |  |
|  |  |  |  |  |  |  |  |  |  |  |  |  |  | 12 |  |  |  |  |  |  |  |
|  |  |  |  |  |  |  |  |  |  |  |  |  |  |  |  |  |  |  |  |  |  |
|  | **RH30** | 12 13 | 11 | 10 | 12 | 17 18 | 9 9.3 | X Y | 8 11 | 10 11 |  |  |  |  |  |  |  | NO |  |  |  |
|  | **RH41** | 13 13 | 8 9 | 10 11 | 12 13 | 16 18 | 7 9.3 | X X | 8 11 | 11 12 | 17 17 | 29 | 15 | 11 | 9 12 | 13 13 | 20 22 | NO |  |  |  |
|  |  |  |  |  |  |  |  |  |  |  |  | 31 | 16 | 17 |  |  |  |  |  |  |  |
|  | **U2OS** | 8 11 | 13 13 | 11 12 | 11 12 | 14 18 | 6 9.3 | X X | 11 12 | 12 13 | 15 16 | 31 | 12 | 10 | 9 | 12 | 20 | NO |  |  |  |
|  |  |  |  |  |  |  |  |  |  |  |  | 31 | 14 | 13 | 9 | 14 | 20 |  |  |  |  |
|  | **Dbt-MYCN** | 10 12 | 8 11 | 8 10 | 10 13 | 16 17 | 9.3 9.3 |  | 9 9 | 10 12 | 15 16 | 28 31 | 13 15 |  |  | 11 13 | 20 26 |  |  |  |  |
|  | **HeLa** | 11 12 | 12 13.3 | 8 12 | 9 10 | 16 18 | 7 | X | 8 12 | 9 10 |  |  |  |  |  |  |  |  |  |  |  |
|  | **HEK293T** | 8 9 | 11 12 15 | 10 11 | 9 12 13 | 16 18 19 20 | 7 9.3 | X X | 11 11 | 10 12 | 16 16 | 28 30.2 | 17 18 19 | 7 15 | 9 10 | 11 12 13 14 | 21 23 | NO |  |  |  |
|  |  |  |  |  |  |  |  |  |  |  |  |  |  |  |  |  |  |  |  |  |  |
| **Mouse cell lines** | **Sample Name** | **M18-3** | **M4-2** | **M6-7** | **M19-2** | **M1-2** | **M7-1** | **M1-1** | **M3-2** | **M8-1** | **M2-1** | **M15-3** | **M6-4** | **M11-2** | **M17-2** | **M12-1** | **M5-5** | **MX-1** | **M13-1** | **D8S1106** | **D4S2408** |
|  | **U37125** | 17 | 17.3 | 12 15 | 13 | 13 | 27.2 | 17 | 14 | 16 | 16 | 20.3 21.3 | 18 | 16 | 16 | 17 | 17 | 26 | 17 | Not Detected | Not Detected |
|  | **U66788** | 17 | 21.3 | 16 | 12 13 | 18 19 | 22.2 23.2 | 10 16 | 14 | 15 17 | 16 | 20.3 22.3 | 17 | 16 17 | 13 | 16 18 | 14 | 26 | 15.2 17 | Not Detected | Not Detected |
|  | **U48484** | 16 | 18.3 | 15 16 | 13 | 13 19 | 23.2 27.2 | 11 15 | 14 | 15 15.3 | 16 | 20.3 23.3 | 15.3 17 | 17 | 13 15 | 16 17 | 14 | 26 | 15.2 17 | Not Detected | Not Detected |
|  | **U57810** | 16 17 | 20.3 21.3 | 12 16 | 12 | 13 | 23.2 | 10 | 14 | 15 17 | 9 16 | 20.3 25.3 | 16 18 | 16 17 | 13 14 | 17 | 15 | 27 | 17 |  |  |
|  | **K7M2** | 18 | 21.3 | 12 | 13 | 17 | 29 | 16 | 12 13 | 13 | 16 | 22.3 23.3 | 18 | 18 | 16 17 | 17 | 14 | 25 | 16.2 17.2 |  |  |
|  | **K12** | 17 18 | 21.3 | 12 | 13 | 17 18 | 29 | 16 | 13 14 | 13 | 16 | 22.3 23.3 | 17 18 | 18 | 16 | 16 17 | 14 | 25 | 16.2 17.2 |  |  |

**Supplementary Table 1.** Fingerprinting of cell lines used in the study.

**Supplementary Table 2.** Primary hits from Figure 2A that were advanced to functional evaluation experiment.

| **Common Name** | **IUPAC Name** | **Molecular Formula** | **Molecular Weight** | **Fold Binding** |
| --- | --- | --- | --- | --- |
| carboplatin (NSC241240) | cis-Diammine(1,1-cyclobutanedicarboxylato)platinum(II) | C_6_H_14_N_2_O_4_Pt | 373.27 | 6.19 |
| dasatinib (NSC732517) | N-(2-chloro-6-methylphenyl)-2-[[6-[4-(2-hydroxyethyl)piperazin-1-yl]-2-methylpyrimidin-4-yl]amino]-1,3-thiazole-5-carboxamide | C_22_H_26_ClN_7_O_2_S | 488.0 | 5.64 |
| lenvatinib (NSC755980) | 4-[3-chloro-4-(cyclopropylcarbamoylamino)phenoxy]-7-methoxyquinoline-6-carboxamide | C_21_H_19_ClN_4_O_4_ | 426.9 | 42.29 |
| rucaparib phosphate (NSC756644) | 6-fluoro-2-[4-(methylaminomethyl)phenyl]-3,10-diazatricyclo[6.4.1.04,13]trideca-1,4,6,8(13)-tetraen-9-one;phosphoric acid | C_19_H_21_FN_3_O_5_P | 421.4 | 5.03 |
| idarubicin hydrochloride (NSC256439) | (7S,9S)-9-acetyl-7-[(2R,4S,5S,6S)-4-amino-5-hydroxy-6-methyloxan-2-yl]oxy-6,9,11-trihydroxy-8,10-dihydro-7H-tetracene-5,12-dione;hydrochloride | C_26_H_28_ClNO_9_ | 534.0 | 8.42 |
| irinotecan hydrochloride (NSC616348) | [(19S)-10,19-diethyl-19-hydroxy-14,18-dioxo-17-oxa-3,13-diazapentacyclo[11.8.0.02,11.04,9.015,20]henicosa-1(21),2,4(9),5,7,10,15(20)-heptaen-7-yl] 4-piperidin-1-ylpiperidine-1-carboxylate;hydrochloride | C_33_H_39_ClN_4_O_6_ | 623.1 | 10.08 |
| NSC34210 | *N*-butan-2-yl-3-methylbutanamide | C_9_H_19_NO | 157.0 | 5.75 |
| tris-p-tolylsulfonium (NSC157930) | tris(4-methylphenyl)sulfanium | C_21_H_21_S+ | 305.5 | 5.83 |
| NSC60530 | triazolo[5,1-f][1,2,4]triazine-4,6-diamine | C_4_H_5_N_7_ | 151.0 | 12.20 |
| sulisobenzonum (NSC60548) | 2-(4-hydroxy-2-oxo-3H-1,3-thiazol-5-yl)acetic acid | C_5_H_5_NO_4_S | 175.0 | 12.05 |
| NSC63314 | 5-ethyl-2-[(*E*)-2-methylpent-1-enyl]pyridine | C_13_H_19_N | 189.0 | 10.59 |
| 2-benzoylpyrrole  (NSC75585) | phenyl(1*H*-pyrrol-2-yl)methanone | C_11_H_9_NO | 171.0 | 7.98 |
| 1-phenyl-5-aminopyrazole (NSC75786) | 2-phenylpyrazol-3-amine | C_9_H_9_N_3_ | 159.0 | 12.34 |
| NSC80807 | (*Z*)-4-oxo-4-(1,3-thiazol-2-ylamino)but-2-enoic acid | C_7_H_6_N_2_O_3_S | 198.0 | 9.42 |
| NSC83345 | 4-phenyl-4,5,6,7-tetrahydro-1*H*-imidazo[4,5-c]pyridine | C_12_H_13_N_3_ | 199.0 | 10.47 |
| NSC84200 | 3-[(dimethylamino)methyl]bicyclo[2.2.1]heptan-2-ol | C_10_H_19_NO | 169.0 | 26.87 |
| NSC299187 | 1-hydroxy-4-[2-(2-hydroxyethylamino)ethylamino]anthracene-9,10-dione | C_18_H_18_N_2_O_4_ | 326.0 | 20.27 |
| NSC109813 | 2,5,8-trimethyl-1*H*-quinolin-4-one | C_12_H_13_NO | 187.0 | 14.61 |
| NSC2805 | 2-(2,5-dihydroxy-4-methylphenyl)-5-methylbenzene-1,4-diol | C_14_H_14_O_4_ | 246.0 | 12.38 |
| NSC3001 | 2-[1-(carboxymethyl)-3-methylcyclohexyl]acetic acid | C_11_H_18_O_4_ | 214.0 | 14.50 |
| NSC8179 | (5-phenyl-1*H*-1,2,4-triazol-3-yl)urea | C_9_H_9_N_5_O | 203.0 | 37.42 |
| NSC8481 | 2-(4-*tert*-butylphenoxy)acetic acid | C_12_H_16_O_3_ | 208.0 | 28.82 |
| NSC10416 | 9-Phenylcarbazole | C_18_H_13_N | 243.0 | 19.95 |
| quinacrine dihydrochloride (NSC14229) | 4-*N*-(6-chloro-2-methoxyacridin-9-yl)-1-*N*,1-*N*-diethylpentane-1,4-diamine;dihydrochloride | C_23_H_32_Cl_3_N_3_O | 473.0 | 8.50 |
| NSC513815 | 5-pyridin-3-yl-1,3,4-thiadiazol-2-amine | C_7_H_6_N_4_S | 178.0 | 18.72 |
| NSC660300 | 1-(2-amino-4-nitrophenyl)piperidine-2,6-dione | C_11_H_11_N_3_O_4_ | 249.0 | 33.47 |
| NSC211336 | *N*-[3-(2-chloro-4-nitrophenoxy)propyl]acetamide | C_11_H_13_ClN_2_O_4_ | 273.0 | 13.78 |
| NSC305329 | 3-(benzimidazol-2-ylideneamino)-2*H*-isoindol-1-ol | C_15_H_10_N_4_O | 262.0 | 35.59 |
| NSC623093 | 2,2,2-trifluoro-*N*-[2-[5-oxo-2,4-bis(phenylcarbamothioyl)-3-sulfanylidene-1,2,4-triazin-6-yl]phenyl]acetamide | C_25_H_17_F_3_N_6_O_2_S_3_ | 587.0 | 8.59 |
| NSC27305 | 9-[6-(hydroxymethyl)-2,2-dimethyl-3*a*,4,6,6*a*-tetrahydrofuro[3,4-d][1,3]dioxol-4-yl]-3*H*-purine-6-thione | C_13_H_16_N_4_O_4_S | 324.0 | 7.55 |
| NSC629659 | 2-(1,3-benzothiazol-2-yl)-6-phenyl-4,5-dihydropyridazin-3-one | C_17_H_13_N_3_OS | 307.0 | 18.34 |
| NSC61642 | 6,7-bis(4-aminophenyl)pteridine-2,4-diamine | C_18_H_16_N_8_ | 344.0 | 6.07 |
| boldine (NSC65689) | (6*aS*)-1,10-dimethoxy-6-methyl-5,6,6*a*,7-tetrahydro-4*H*-dibenzo[de,g]quinoline-2,9-diol | C_19_H_21_NO_4_ | 327.0 | 5.11 |
| NSC167410 | 2-(3,4-dihydroxyphenyl)-6,8-dihydroxy-3-(4,5,6-trihydroxy-3-methyloxan-2-yl)oxychromen-4-one | C_21_H_20_O_11_ | 448.0 | 53.29 |
| hydroberberubin  (NSC123389) | 16-methoxy-5,7-dioxa-13-azapentacyclo[11.8.0.02,10.04,8.015,20]henicosa-2,4(8),9,15(20),16,18-hexaen-17-ol | C_19_H_19_NO_4_ | 325.0 | 7.65 |
| methergine (NSC186067) | *N*-(1-hydroxybutan-2-yl)-7-methyl-6,6*a*,8,9-tetrahydro-4*H*-indolo[4,3-fg]quinoline-9-carboxamide | C_20_H_25_N_3_O_2_ | 339.0 | 11.36 |
| NSC632536 | 4-*N*-phenyl-1-*N*-(2,2,6,6-tetramethylpiperidin-4-yl)benzene-1,4-diamine | C_21_H_29_N_3_ | 323.0 | 8.59 |
| NSC204232 | 1-*N*,3-*N*-bis(3-nitrophenyl)benzene-1,3-dicarboxamide | C_20_H_14_N_4_O_6_ | 406.0 | 36.04 |
| 9-aminocamptothecin (NSC603071) | (19*S*)-8-amino-19-ethyl-19-hydroxy-17-oxa-3,13-diazapentacyclo[11.8.0.02,11.04,9.015,20]henicosa-1(21),2,4,6,8,10,15(20)-heptaene-14,18-dione | C_20_H_17_N_3_O_4_ | 363.0 | 35.32 |
| polyoxomolybdate phosphate complex, disodium salt  (NSC622116) |  | HMo_12_Na_2_O_40_P | 1869.0 | 9.09 |
| quinic acid (NSC1115) | (3*S*,5*S*)-1,3,4,5-tetrahydroxycyclohexane-1-carboxylic acid | C_7_H_12_O_6_ | 192.0 | 5.17 |
| aristolochic acid (NSC11926) | 8-methoxy-6-nitronaphtho[2,1-g][1,3]benzodioxole-5-carboxylic acid | C_17_H_11_NO_7_ | 341.0 | 3.46 |
| xanthine (NSC14664) | 3,7-dihydropurine-2,6-dione | C_5_H_4_N_4_O_2_ | 152.0 | 5.84 |
| deuteroporphyrin (NSC18298) | 3-[18-(2-carboxyethyl)-3,8,13,17-tetramethyl-22,23-dihydroporphyrin-2-yl]propanoic acid | C_30_H_30_N_4_O_4_ | 572.0 | 77.44 |
| aspergillic acid (NSC22939) | 6-butan-2-yl-1-hydroxy-3-(2-methylpropyl)pyrazin-2-one | C_12_H_20_N_2_O_2_ | 224.0 | 6.16 |
| (-)-cephaeline dihydrochloride  (NSC32944) | (1*R*)-1-[[(2*S*,3*R*,11*bS*)-3-ethyl-9,10-dimethoxy-2,3,4,6,7,11*b*-hexahydro-1*H*-benzo[a]quinolizin-2-yl]methyl]-7-methoxy-1,2,3,4-tetrahydroisoquinolin-6-ol;dihydrochloride | C_28_H_40_Cl_2_N_2_O_4_ | 540.0 | 7.41 |
| NSC96932 | (2*Z*)-3-ethyl-2-[(*E*)-3-(3-ethyl-1,3-benzothiazol-3-ium-2-yl)-2-methylprop-2-enylidene]-1,3-benzothiazole;iodide | C_22_H_23_IN_2_S_2_ | 506.0 | 7.57 |
| isolupinine, (D), N-oxide  (NSC34552) | (5-oxido-2,3,4,6,7,8,9,9*a*-octahydro-1*H*-quinolizin-5-ium-4-yl)methanol | C_10_H_19_NO_2_ | 185.0 | 5.03 |
| 3,4-dihydropapaverine oxalate (NSC35550) | 1-[(3,4-dimethoxyphenyl)methyl]-6,7-dimethoxy-3,4-dihydroisoquinoline;oxalic acid | C_22_H_25_NO_8_ | 431.0 | 5.02 |
| NSC56737 | *N*-[(*E*)-(3-methoxyphenyl)methylideneamino]-2-[1-[(2*Z*)-2-[(3-methoxyphenyl)methylidene]hydrazinyl]-1-oxopropan-2-yl]sulfanylpropanamide | C_22_H_26_N_4_O_4_S | 443.0 | 21.00 |
| pseudotropine (NSC43871) | (1*S*,5*R*)-8-methyl-8-azabicyclo[3.2.1]octan-3-ol | C_8_H_15_NO | 141.0 | 6.20 |
| thapsine acetate (NSC76022) | acetic acid;5-[2-(dimethylamino)ethyl]-7,14-dimethoxy-2,9-dioxatetracyclo[6.6.2.04,16.011,15]hexadeca-1(14),4(16),5,7,11(15),12-hexaene-3,10-dione | C_22_H_23_NO_8_ | 429.4 | 8.65 |
| NSC169676 | 2-[2-[4-[3-[2-(trifluoromethyl)phenothiazin-10-yl]propyl]piperazin-1-yl]ethylamino]ethanol;trihydrochloride | C_24_H_34_Cl_3_F_3_N_4_OS | 590.0 | 6.34 |
| echinatine (NSC89937) | [(7*S*,8*R*)-7-hydroxy-5,6,7,8-tetrahydro-3*H*-pyrrolizin-1-yl]methyl (2*S*)-2-hydroxy-2-[(1*S*)-1-hydroxyethyl]-3-methylbutanoate | C_15_H_25_NO_5_ | 299.0 | 5.41 |
| NSC177365 | 2-[3-[[4-[(3-nitroacridin-9-yl)amino]phenyl]sulfamoyl]propyl]guanidine | C_23_H_23_N_7_O_4_S | 566.0 | 49.12 |
| NSC316157 | 1,4-dihydroxy-2-[2-(2-hydroxyethylamino)ethylamino]anthracene-9,10-dione | C_18_H_18_N_2_O_5_ | 342.0 | 16.16 |
| NSC335142 | 5,11-dimethyl-6*H*-pyrido[4,3-b]carbazole-1-carboxamide;hydrochloride | C_18_H_16_ClN_3_O | 326.0 | 14.37 |
| NSC624169 | 2-[[2-[(2-aminoethyldisulfanyl)methyl]phenyl]methyldisulfanyl]ethanamine;hydrochloride | C_12_H_21_ClN_2_S_4_ | 393.0 | 10.37 |
| NSC288010 | 1-(4-chlorophenyl)-*N*-[2-(diethylamino)ethyl]-7-methoxy-4,5-dihydrobenzo[g]indazole-3-carboxamide;hydrochloride | C_25_H_30_Cl_2_N_4_O_2_ | 489.0 | 10.36 |
| kasuagamycin (NSC100858) | 1-(4-chlorophenyl)-*N*-[2-(diethylamino)ethyl]-7-methoxy-4,5-dihydrobenzo[g]indazole-3-carboxamide;hydrochloride | C_14_H_25_N_3_O_9_ | 379.0 | 5.36 |
| pinaverium bromide | 4-[(2-bromo-4,5-dimethoxyphenyl)methyl]-4-[2-[2-(6,6-dimethyl-4-bicyclo[3.1.1]heptanyl)ethoxy]ethyl]morpholin-4-ium bromide | C_26_H_41_Br_2_NO_4_ | 591.4 | 23.67 |
| irinotecan hydrochloride trihydrate | (S)-4,11-diethyl-3,4,12,14-tetrahydro-4-hydroxy-3,14-dioxo1H-pyrano[3’,4’:6,7]-indolizino[1,2-b]quinolin-9-yl-[1,4’bipiperidine]-1’-carboxylate hydrochloride trihydrate | C_33_H_45_ClN_4_O_9_ | 667.2 | 40.17 |
| pantoprazole sodium | 5-(difluoromethoxy)-2-[(3,4-dimethoxypyridin-2-yl)methylsulfinyl]benzimidazol-1-ide sodium | C_16_H_14_F_2_N_3_NaO_4_S | 405.4 | 6.19 |
| acetylsalicylsalicylic acid | 2-(2-acetyloxybenzoyl)oxybenzoic acid | C_16_H_12_O_6_ | 300.3 | 8.81 |
| R(-) apomorphine hydrochloride hemihydrate | (6aR)-6-Methyl-5,6,6a,7-tetrahydro-4H-dibenzo[de,g]quinoline-10,11-diol hydrochloride hydrate | C_34_H_38_Cl_2_N_2_O_5_ | 625.6 | 8.86 |
| didanosine | 9-[(2R,5S)-5-(hydroxymethyl)oxolan-2-yl]-3H-purin-6-one | C_10_H_12_N_4_O_3_ | 236.2 | 6.70 |
| erlotinib | N-(3-Ethynylphenyl)-6,7-bis(2-methoxyethoxy)-4-quinazolinamine | C_22_H_23_N_3_O_4_ | 393.5 | 5.76 |
| hydroxyacrine maleate (R,S) | 9-amino-1,2,3,4-tetrahydroacridin-4-ol (Z)-but-2-enedioic acid | C_17_H_18_N_3_O_5_ | 330.3 | 22.30 |
| levamisole hydrochloride | (6S)-6-phenyl-2,3,5,6-tetrahydroimidazo[2,1-b][1,3]thiazole hydrochloride | C_11_H_13_ClN_2_S | 240.8 | 24.07 |
| meclofenamic acid sodium salt monohydrate | 2-[(2,6-Dichloro-3-methylphenyl)amino]benzoic acid sodium salt | C_14_H_12_Cl_2_NNaO_3_ | 336.2 | 5.02 |
| hycanthone | 1-(2-Diethylaminoethylamino)-4-(hydroxymethyl)-9-thioxanthenone | C_20_H_24_N_2_O_2_S | 356.5 | 7.47 |
| methylergometrine maleate | (6aR,9R)-N-[(2S)-1-hydroxybutan-2-yl]-7-methyl-6,6a,8,9-tetrahydro-4H-indolo[4,3-fg]quinoline-9-carboxamide (Z)-but-2-enedioate | C_24_H_29_N_3_O_6_ | 455.5 | 7.57 |
| amikacin hydrate | (2S)-4-Amino-N-{(1R,2S,3S,4R,5S)-5-amino-2-[(3-amino-3-deoxy-alpha-D-glucopyranosyl)oxy]-4-[(6-amino-6-deoxy-alpha-D-glucopyranosyl)oxy]-3-hydroxycyclohexyl}-2-hydroxybutanamide | C_22_H_47_N_5_O_15_ | 621.6 | 7.56 |
| prochlorperazine dimaleate | 2-chloro-10-[3-(4-methylpiperazin-1-yl)propyl]phenothiazine (Z)-but-2-enedioic acid | C_28_H_32_CIN_3_O_8_S | 606.1 | 14.71 |
| zuclopenthixol dihydrochloride | 2-[4-[(3Z)-3-(2-chlorothioxanthen-9-ylidene)propyl]piperazin-1-yl]ethanol dihydrochloride | C_22_H_27_Cl_3_N_3_O_8_S | 473.9 | 6.41 |
| sertraline | (1S,4S)-4-(3,4-dichlorophenyl)-N-methyl-1,2,3,4-tetrahydronaphthalen-1-amine | C_17_H_17_Cl_3_N | 306.2 | 7.31 |
| alcuronium chloride | 4,4'-Didemethyl-4,4'-di-propenyltoxiferin-1-dichloride | C_44_H_50_Cl_3_N_4_O_2_ | 737.8 | 13.87 |
| sulfasalazine | 2-hydroxy-5-[(E)-2-{4-[(pyridin-2-yl)sulfamoyl]phenyl}diazen-1-yl]benzoic acid | C_18_H_14_N_4_O_5_S | 398.4 | 6.83 |
| tobramycin | (2S,3R,4S,5S,6R)-4-amino-2-[(1S,2S,3R,4S,6R)-4,6-diamino-3-[(2R,3R,5S,6R)-3-amino-6-(aminomethyl)-5-hydroxyoxan-2-yl]oxy-2-hydroxycyclohexyl]oxy-6-(hydroxymethyl)oxane-3,5-diol | C_18_H_37_N_5_O_9_ | 467.5 | 37.73 |
| paroxetine hydrochloride | (3S,4R)-3-(1,3-benzodioxol-5-yloxymethyl)-4-(4-fluorophenyl)piperidine hydrochloride | C_19_H_21_ClFNO_9_ | 365.8 | 11.49 |
| liothyronine | (2S)-2-amino-3-[4-(4-hydroxy-3-iodophenoxy)-3,5-diiodophenyl]propanoic acid | C_15_H_12_I_3_NO_4_ | 650.9 | 13.70 |
| demecarium bromide | trimethyl-[3-[methyl-[10-[methyl-[3-(trimethylazaniumyl)phenoxy]carbonylamino]decyl]carbamoyl]oxyphenyl]azanium dibromide | C_32_H_52_Br_2_N_4_O_4_ | 716.6 | 5.72 |
| bephenium hydroxynaphthoate | benzyl-dimethyl-(2-phenoxyethyl)azanium 3-carboxynaphthalen-2-olate | C_28_H_29_NO_4_ | 443.6 | 7.03 |
| benserazide hydrochloride | 3-[[6-(3-carboxy-2,4,6-triiodoanilino)-6-oxohexanoyl]amino]-2,4,6-triiodobenzoic acid | C_20_H_14_I_6_N_2_O_6_ | 1139.8 | 5.59 |
| salmeterol | 2-(hydroxymethyl)-4-[1-hydroxy-2-[6-(4-phenylbutoxy)hexylamino]ethyl]phenol | C_25_H_37_NO_4_ | 415.6 | 6.66 |
| prazosin hydrochloride | [4-(4-amino-6,7-dimethoxyquinazolin-2-yl)piperazin-1-yl]-(furan-2-yl)methanone hydrochloride | C_19_H_21_ClN_5_O_4_ | 419.9 | 9.71 |
| stavudine | 1-[(2R,5S)-5-(hydroxymethyl)-2,5-dihydrofuran-2-yl]-5-methylpyrimidine-2,4-dione | C_10_H_12_N_2_O_4_ | 224.2 | 6.76 |
| megestrol acetate | 17-(acetyloxy)-6-methyl-pregna-4,6-diene-3,20-dione | C_24_H_32_O_4_ | 384.5 | 10.54 |
| imatinib | 4-[(4-methylpiperazin-1-yl)methyl]-N-[4-methyl-3-[(4-pyridin-3-ylpyrimidin-2-yl)amino]phenyl]benzamide | C_29_H_31_N_7_O | 493.6 | 19.01 |
| aclacinomycin A (NSC208734) | methyl (1*R*,2*R*,4*S*)-4-[(2*R*,4*S*,5*S*,6*S*)-4-(dimethylamino)-5-[(2*S*,4*S*,5*S*,6*S*)-4-hydroxy-6-methyl-5-[(2*R*,6*S*)-6-methyl-5-oxooxan-2-yl]oxyoxan-2-yl]oxy-6-methyloxan-2-yl]oxy-2-ethyl-2,5,7-trihydroxy-6,11-dioxo-3,4-dihydro-1*H*-tetracene-1-carboxylate | C_42_H_53_NO_15_ | 812.0 | 9.88 |
| epirubicin hydrochloride (NSC256942) | (7*S*,9*S*)-7-[(2*R*,4*S*,5*R*,6*S*)-4-amino-5-hydroxy-6-methyloxan-2-yl]oxy-6,9,11-trihydroxy-9-(2-hydroxyacetyl)-4-methoxy-8,10-dihydro-7*H*-tetracene-5,12-dione;hydrochloride | C_27_H_30_ClNO_11_ | 580.0 | 6.00 |
| NSC332294 | (6*aR*,9*R*,10*aR*)-7,9-dimethyl-4-propyl-6,6*a*,8,9,10,10*a*-hexahydroindolo[4,3-fg]quinoline;(2*R*,3*R*)-2,3-dihydroxybutanedioic acid | C_23_H_32_N_2_O_6_ | 433.0 | 5.95 |
| albacarcin V (NSC354844) | (6*aR*,9*R*,10*aR*)-7,9-dimethyl-4-propyl-6,6*a*,8,9,10,10*a*-hexahydroindolo[4,3-fg]quinoline;(2*R*,3*R*)-2,3-dihydroxybutanedioic acid | C_28_H_28_O_9_ | 509.0 | 62.87 |
| rebeccamycin (NSC359079) | 5,21-dichloro-3-[(2*R*,3*R*,4*R*,5*S*,6*R*)-3,4-dihydroxy-6-(hydroxymethyl)-5-methoxyoxan-2-yl]-3,13,23-triazahexacyclo[14.7.0.02,10.04,9.011,15.017,22]tricosa-1,4(9),5,7,10,15,17(22),18,20-nonaene-12,14-dione | C_27_H_21_Cl_2_N_3_O_7_ | 570.0 | 16.32 |
| imerubrine (NSC785144) | 5,14,15,16-tetramethoxy-10-azatetracyclo[7.7.1.02,8.013,17]heptadeca-1(16),2,4,7,9,11,13(17),14-octaen-6-one | C_20_H_17_NO_5_ | 351.4 | 6.85 |
| homomoschatoline (NSC785149) | 14,15,16-trimethoxy-10-azatetracyclo[7.7.1.02,7.013,17]heptadeca-1(16),2,4,6,9,11,13(17),14-octaen-8-on | C_19_H_15_NO_4_ | 321.3 | 5.92 |
| dehydroanonaine (NSC785156) | 3,5-dioxa-11-azapentacyclo[10.7.1.02,6.08,20.014,19]icosa-1(20),2(6),7,12,14,16,18-heptaene | C17H13NO2 | 263.3 | 7.30 |
| isoboldine (NSC785158) | (6*aS*)-2,10-dimethoxy-6-methyl-5,6,6*a*,7-tetrahydro-4*H*-dibenzo[de,g]quinoline-1,9-diol | C_19_H_21_NO_4_ | 327.4 | 6.86 |
| nandigerine (NSC785160) | (12*S*)-18-methoxy-3,5-dioxa-11-azapentacyclo[10.7.1.02,6.08,20.014,19]icosa-1(20),2(6),7,14(19),15,17-hexaen-17-ol | C_18_H_17_NO_4_ | 311.3 | 7.18 |
| O-methylbulbocapnine (NSC785162) | (12*S*)-17,18-dimethoxy-11-methyl-3,5-dioxa-11-azapentacyclo[10.7.1.02,6.08,20.014,19]icosa-1(20),2(6),7,14(19),15,17-hexaene | C_20_H_21_NO_4_ | 339.4 | 5.01 |
| O-methylsciadenine (NSC785174) | (11*R*,26*S*)-4,5,19,20-tetramethoxy-10,25-dimethyl-2,17-dioxa-10,25-diazaheptacyclo[26.2.2.213,16.13,7.118,22.011,36.026,33]hexatriaconta-1(31),3(36),4,6,13,15,18(33),19,21,28(32),29,34-dodecaene | C_38_H_42_N_2_O_6_ | 622.7 | 12.12 |
| N-methyl hernangerin (NSC785182) | (12*S*)-18-methoxy-11-methyl-3,5-dioxa-11-azapentacyclo[10.7.1.02,6.08,20.014,19]icosa-1(20),2(6),7,14(19),15,17-hexaen-17-ol | C_19_H_19_NO_4_ | 325.4 | 10.28 |
| grisabine (NSC785183) | (1*R*)-1-[[4-[5-[[(1*S*)-7-hydroxy-6-methoxy-2-methyl-3,4-dihydro-1*H*-isoquinolin-1-yl]methyl]-2-methoxyphenoxy]phenyl]methyl]-6-methoxy-2-methyl-3,4-dihydro-1*H*-isoquinolin-7-ol | C_37_H_42_N_2_O_6_ | 610.7 | 10.68 |
| 1,10-dihydroxy-2-methoxyaporphine (NSC785186) | 2-methoxy-6-methyl-5,6,6*a*,7-tetrahydro-4*H*-dibenzo[de,g]quinoline-1,10-diol | C_18_H_19_NO_3_ | 297.3 | 5.84 |
| N-methyllaurotetanine (NSC785189) | (6*aS*)-1,2,10-trimethoxy-6-methyl-5,6,6*a*,7-tetrahydro-4*H*-dibenzo[de,g]quinolin-9-ol | C_20_H_23_NO_4_ | 341.4 | 11.52 |
| NSC697468 | 2-[[2-[2-[5-acetamido-3-hydroxy-2-(hydroxymethyl)-6-phenylmethoxyoxan-4-yl]oxypropanoylamino]-3-methylbutanoyl]amino]-*N*'-[4-[(1-nitroacridin-9-yl)amino]butyl]pentanediamide | C_45_H_58_N_8_O_12_ | 903.0 | 17.20 |
| NSC690634 | *N*,*N*'-bis(1-nitroacridin-9-yl)propane-1,3-diamine | C_29_H_22_N_6_O_4_ | 519.0 | 17.76 |
| glycylaminophenylbenzoylurea (HCl salt) (NSC654259) | 2-[(2-aminoacetyl)amino]-*N*-[[4-(5-bromopyrimidin-2-yl)oxy-3-methylphenyl]carbamoyl]benzamide;hydrochloride | C_21_H_20_BrClN_6_O_4_ | 536.0 | 35.85 |
| NSC635833 | methyl 4-(2,3-dimethylanilino)-2,4-dioxo-3-(3-oxo-4*H*-1,4-benzothiazin-2-yl)butanoate | C_21_H_20_N_2_O_5_S | 412.0 | 23.37 |
| NSC698031 | 1-[5-[(*E*)-2-[4-(dimethylamino)phenyl]ethenyl]-6-methyl-1,2,4-triazin-3-yl]-6-hydroxy-3-phenyl-2-sulfanylidenepyrimidin-4-one | C_24_H_22_N_6_O_2_S | 459.0 | 29.31 |
| etomidate | 'ethyl 3-(1-phenylethyl)imidazole-4-carboxylate | C_14_H_16_N_2_O_2_ | 244.3 | 5.51 |
| piperacetazine | 1-[10-[3-[4-(2-hydroxyethyl)piperidin-1-yl]propyl]phenothiazin-2-yl]ethanone | C_24_H_30_N_2_O_2_S | 410.6 | 5.54 |
| metformin hydrochloride | 1-carbamimidamido-N,N-dimethylmethanimidamide | C_4_H_12_ClN_5_ | 165.6 | 6.27 |
| meticrane | 6-methylthiochromane-7-sulfonamide 1,1-dioxide | C_10_H_13_NO_4_S_2_ | 275.4 | 6.06 |
| mefenamic acid | 2-(2,3-dimethylphenyl)aminobenzoic acid | C_15_H_15_NO_2_ | 241.3 | 6.60 |
| sulpiride | (±)-5-(aminosulfonyl)-N-[(1-ethylpyrrolidin-2-yl)methyl]-2-methoxybenzamide | C_15_H_23_N_3_O_4_S | 341.4 | 23.83 |
| albendazole | methyl N-(6-propylsulfanyl-1H-benzimidazol-2-yl)carbamate | C_12_H_15_N_3_O_2_S | 265.3 | 14.61 |
| quinacrine dihydrochloride hydrate | 6-Chloro-9-(4-diethylamino-1-methylbutyl)amino-2-methoxyacridine dihydrochloride hydrate | C_23_H_36_Cl_3_N_3_O_3_ | 508.9 | 5.60 |

**Supplementary Table 3.** Secondary hits identified by the luciferase reporter assay described in Figure 2B.

| **Compound Name** | **Structure** | **% Activity of PAX3::FOXO1 (Relative to Control Treatment)** | **% Activity of PGK (Relative to Control Treatment)** | **% R_max_ PAX3:: FOXO1 (SPR)** | **Fold-Difference PAX3::FOXO1 vs Control Binding (SPR)** |
| --- | --- | --- | --- | --- | --- |
| NSC697468 | 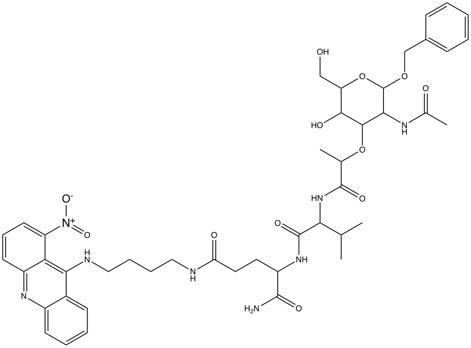 | 0.02823246 | 117.5048 | 137.50 | 17.20 |
| alcuronium chloride | 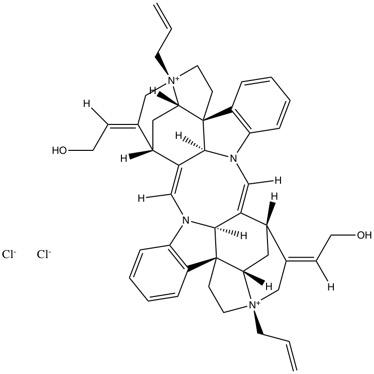 | 15.77796 | 99.78218 | 55.45 | 13.87 |
| NSC2805 | 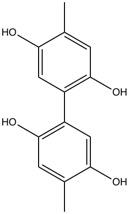 | 20.22917 | 117.8538 | 78.36 | 12.38 |
| didanosine | 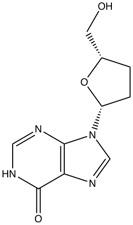 | 15.17979 | 106.8316 | 62.86 | 6.70 |
| carboplatin | 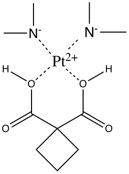 | 0.287854 | 112.4189 | 180.54 | 6.19 |
| piperacetazine | 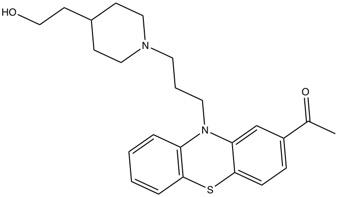 | 28.18571 | 136.0233 | 177.28 | 5.54 |
