# Supplementary material for: Piperacetazine Directly Binds to the PAX3::FOXO1 Fusion Protein and Inhibits Its Transcriptional Activity
Source: Cancer Res Commun. 2023 Oct 6;3(10):2030–43. doi: 10.1158/2767-9764.CRC-23-0119 (PMC10557868; doi:10.1158/2767-9764.CRC-23-0119)
Supplement: Supplementary Table 5 [file crc-23-0119-s03.docx]

**Supplementary Table 5.** Potential activators of PAX3::FOXO1 identified by the secondary screen described in Figure 2B.

| **Compound Name** | **Structure** | **% Activity of PAX3::FOXO1 (Relative to Control Treatment)** | **% Activity of PGK (Relative to Control Treatment)** | **% R_max_ PAX3::FOXO1 (SPR)** | **Fold-Difference PAX3::FOXO1 vs Control Binding (SPR)** |
| --- | --- | --- | --- | --- | --- |
| lauroscholtzine | 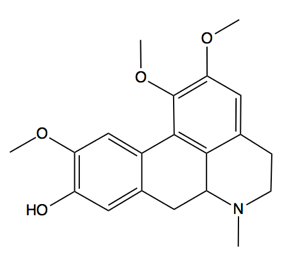 | 628.6205 | 87.89277 | 138.74 | 11.52 |
| hernangerine | 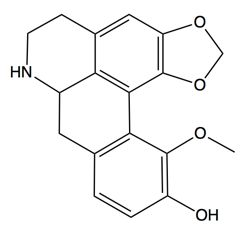 | 567.1904 | 68.46781 | 197.24 | 7.18 |
| imerubrine | 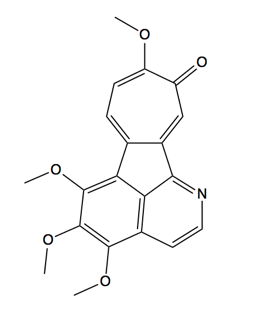 | 574.4699 | 85.92624 | 137.62 | 6.85 |
| aspergillic acid | 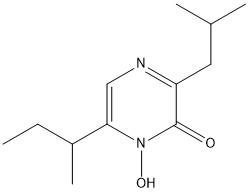 | 546.23 | 94.80737 | 60.65 | 6.16 |
| homomoschatoline | 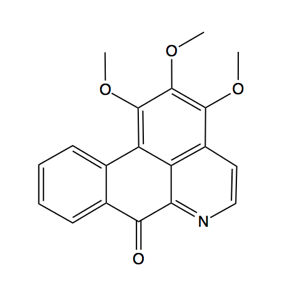 | 524.3458 | 55.46242 | 63.69 | 5.92 |
| xanthine | 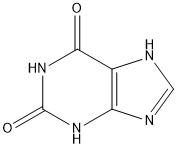 | 533.1564 | 79.67545 | 58.00 | 5.84 |
| erlotinib | 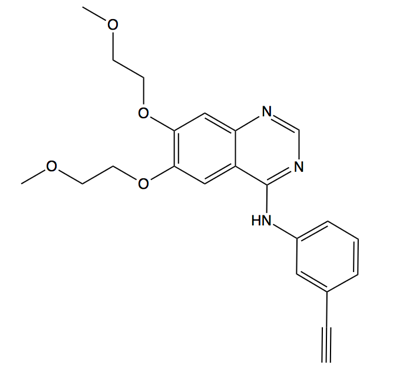 | 516.6344 | 79.31454 | 65.47 | 5.76 |
| etomidate | 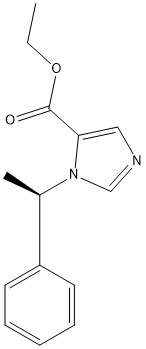 | 580.8079 | 100.7689 | 59.29 | 5.51 |
| echinatine | 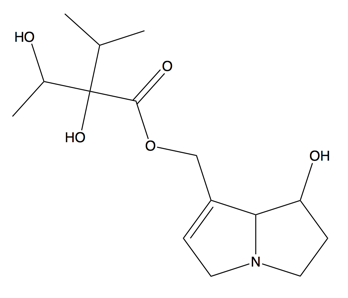 | 517.5983 | 76.69685 | 104.67 | 5.41 |
| kasugamycin | 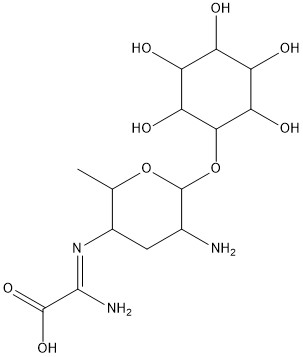 | 547.4194 | 52.94705 | 149.47 | 5.36 |
