# Supplementary material for: Piperacetazine Directly Binds to the PAX3::FOXO1 Fusion Protein and Inhibits Its Transcriptional Activity
Source: Cancer Res Commun. 2023 Oct 6;3(10):2030–43. doi: 10.1158/2767-9764.CRC-23-0119 (PMC10557868; doi:10.1158/2767-9764.CRC-23-0119)
Supplement: Supplementary Figure 1 — Nucleotide and amino acid sequences of PAX3::FOXO1 gene in bacterial expression plasmid pET104.1 are provided. [file crc-23-0119-s04.pptx]

## Slide 1
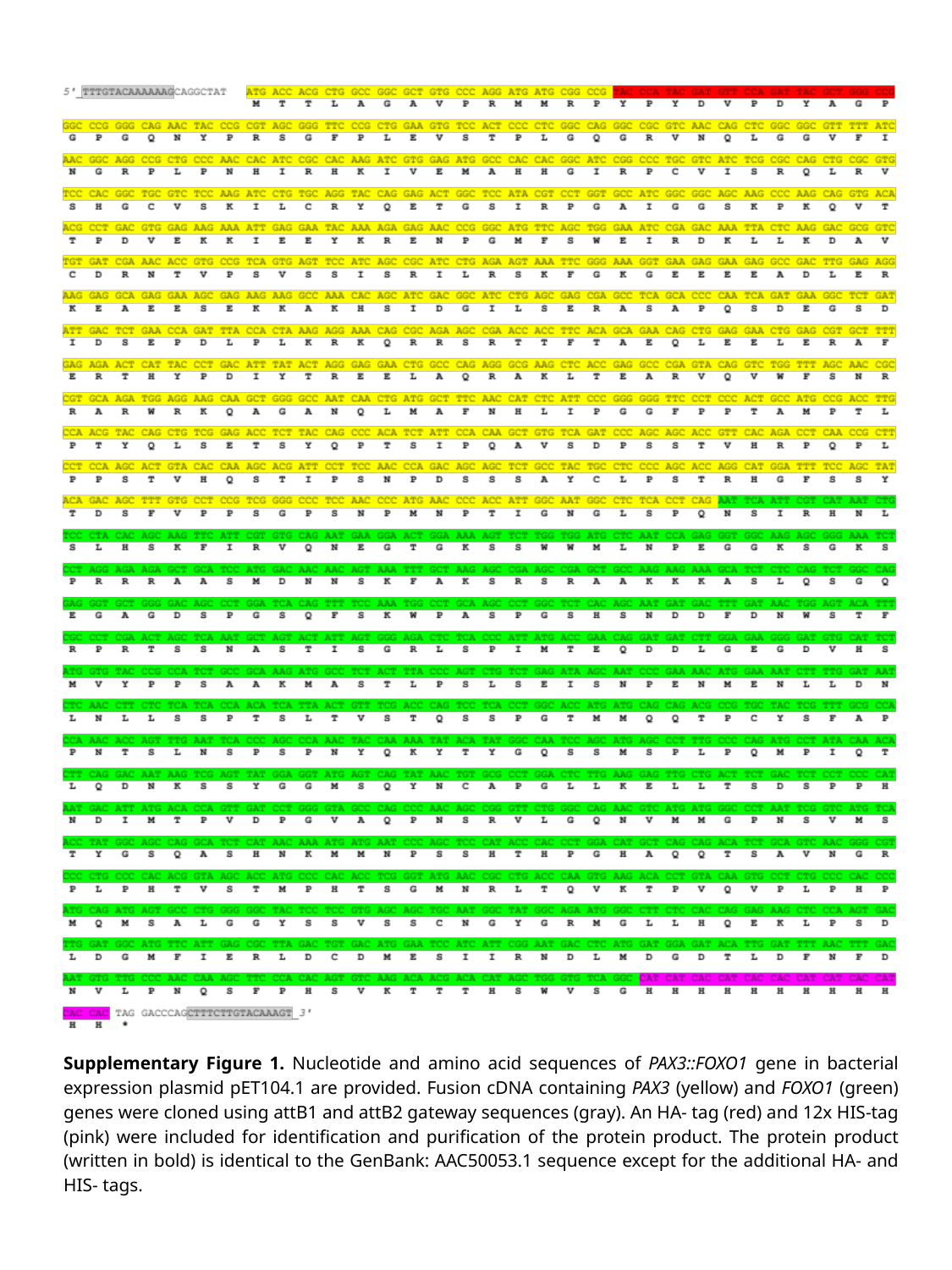

Supplementary Figure 1. Nucleotide and amino acid sequences of PAX3::FOXO1 gene in bacterial expression plasmid pET104.1 are provided. Fusion cDNA containing PAX3 (yellow) and FOXO1 (green) genes were cloned using attB1 and attB2 gateway sequences (gray). An HA- tag (red) and 12x HIS-tag (pink) were included for identification and purification of the protein product. The protein product (written in bold) is identical to the GenBank: AAC50053.1 sequence except for the additional HA- and HIS- tags.
