# Supplementary material for: Piperacetazine Directly Binds to the PAX3::FOXO1 Fusion Protein and Inhibits Its Transcriptional Activity
Source: Cancer Res Commun. 2023 Oct 6;3(10):2030–43. doi: 10.1158/2767-9764.CRC-23-0119 (PMC10557868; doi:10.1158/2767-9764.CRC-23-0119)
Supplement: Supplementary Figure 2 — Luciferase assay performed to measure the effect of piperacetazine on PAX7::FOXO1 activity. [file crc-23-0119-s05.pptx]

## Slide 1
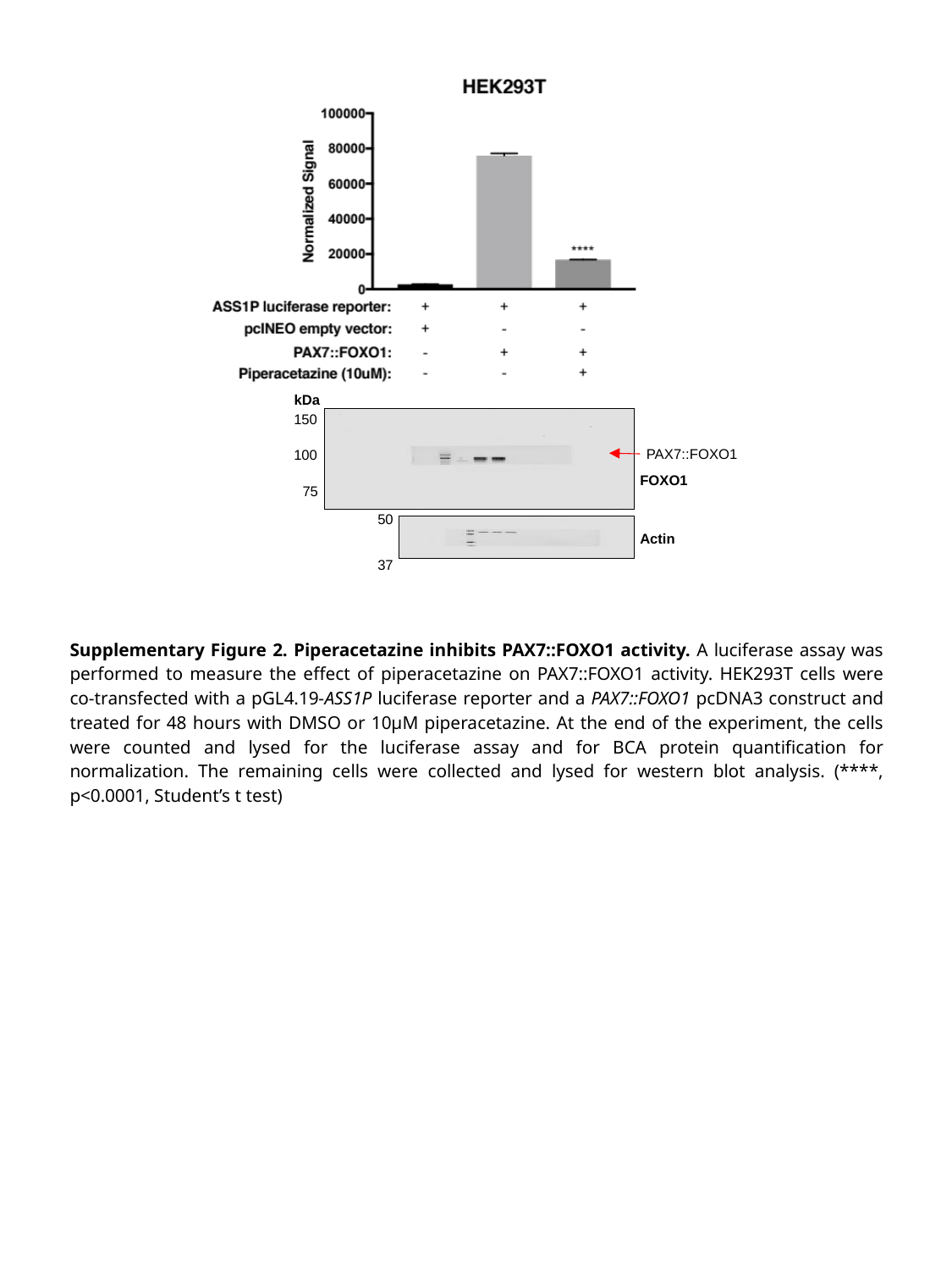

kDa
150
PAX7::FOXO1
100
FOXO1
75
50
Actin
37
Supplementary Figure 2. Piperacetazine inhibits PAX7::FOXO1 activity. A luciferase assay was performed to measure the effect of piperacetazine on PAX7::FOXO1 activity. HEK293T cells were co-transfected with a pGL4.19-ASS1P luciferase reporter and a PAX7::FOXO1 pcDNA3 construct and treated for 48 hours with DMSO or 10μM piperacetazine. At the end of the experiment, the cells were counted and lysed for the luciferase assay and for BCA protein quantification for normalization. The remaining cells were collected and lysed for western blot analysis. (****, p<0.0001, Student’s t test)
