# Supplementary material for: Piperacetazine Directly Binds to the PAX3::FOXO1 Fusion Protein and Inhibits Its Transcriptional Activity
Source: Cancer Res Commun. 2023 Oct 6;3(10):2030–43. doi: 10.1158/2767-9764.CRC-23-0119 (PMC10557868; doi:10.1158/2767-9764.CRC-23-0119)
Supplement: Supplementary Figure 3 — Piperacetazine IC50 values across various cell lines. [file crc-23-0119-s06.pptx]

## Slide 1
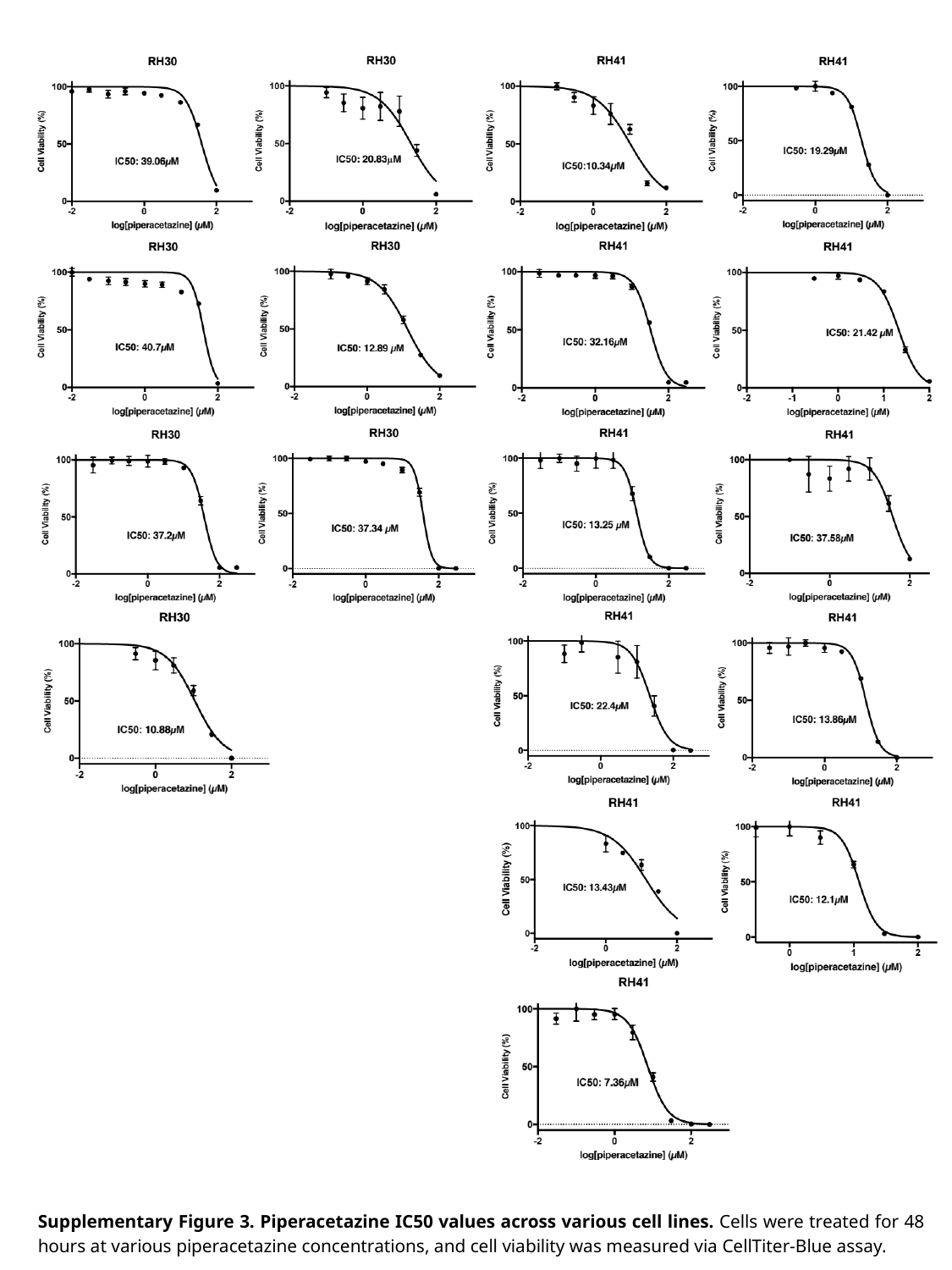

Supplementary Figure 3. Piperacetazine IC50 values across various cell lines. Cells were treated for 48 hours at various piperacetazine concentrations, and cell viability was measured via CellTiter-Blue assay.

## Slide 2
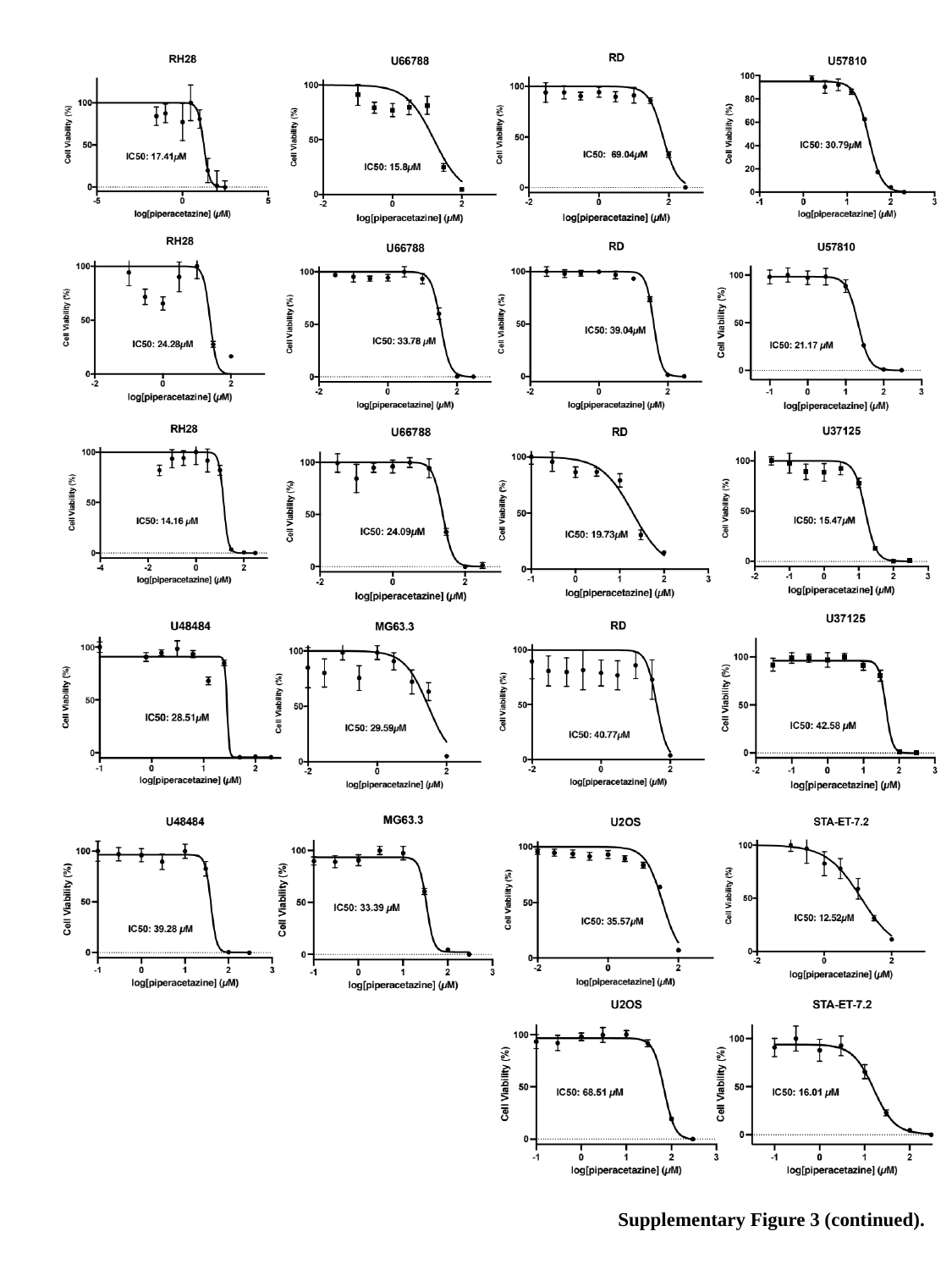

Supplementary Figure 3 (continued).

## Slide 3
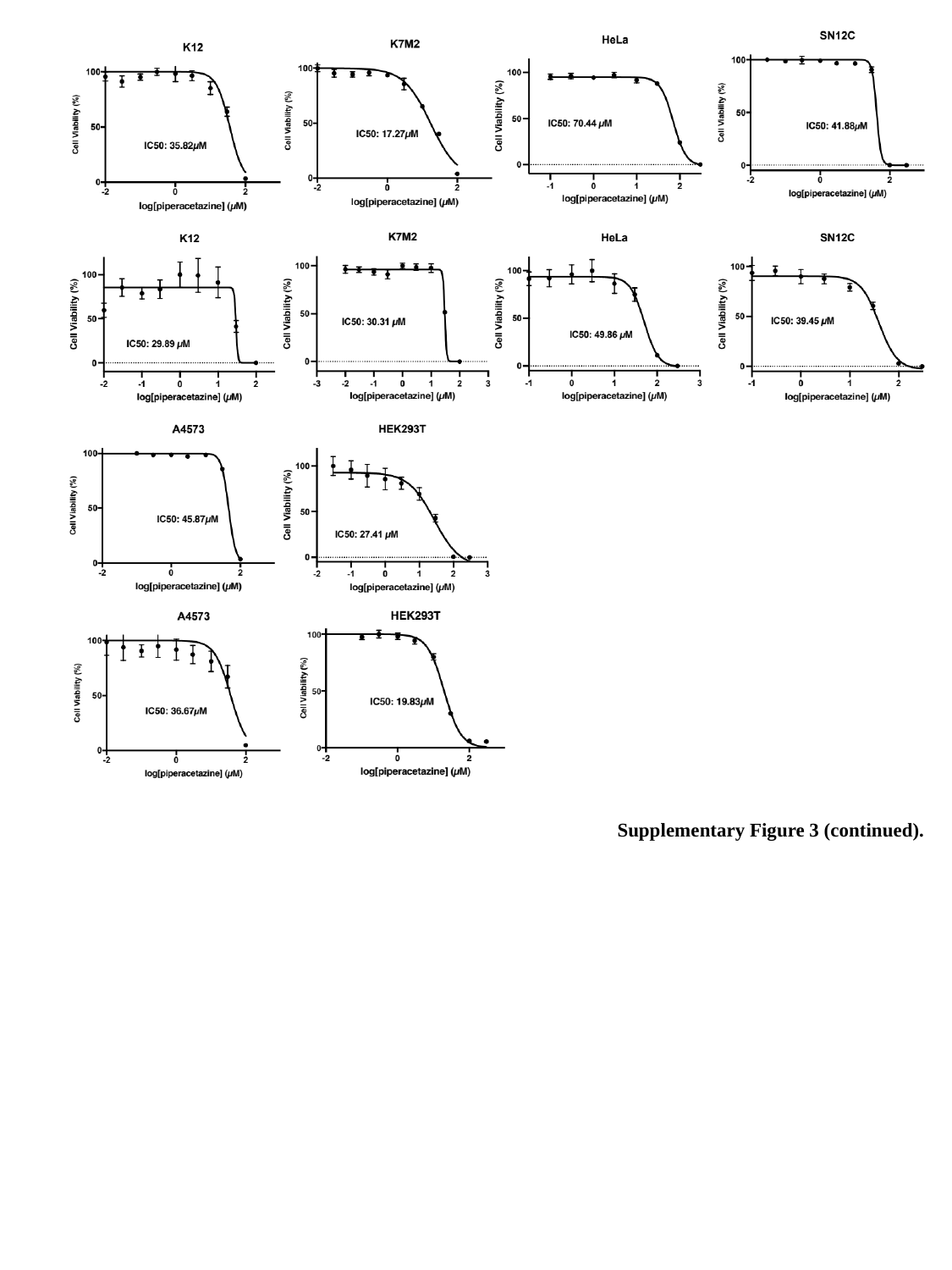

Supplementary Figure 3 (continued).
