# Supplementary material for: Piperacetazine Directly Binds to the PAX3::FOXO1 Fusion Protein and Inhibits Its Transcriptional Activity
Source: Cancer Res Commun. 2023 Oct 6;3(10):2030–43. doi: 10.1158/2767-9764.CRC-23-0119 (PMC10557868; doi:10.1158/2767-9764.CRC-23-0119)
Supplement: Supplementary Figure 4 — Reduction of PAX3::FOXO1 protein expression does not alter proliferation rates of FP-RMS cell lines but reduces anchorage-independent growth. [file crc-23-0119-s07.pptx]

## Slide 1
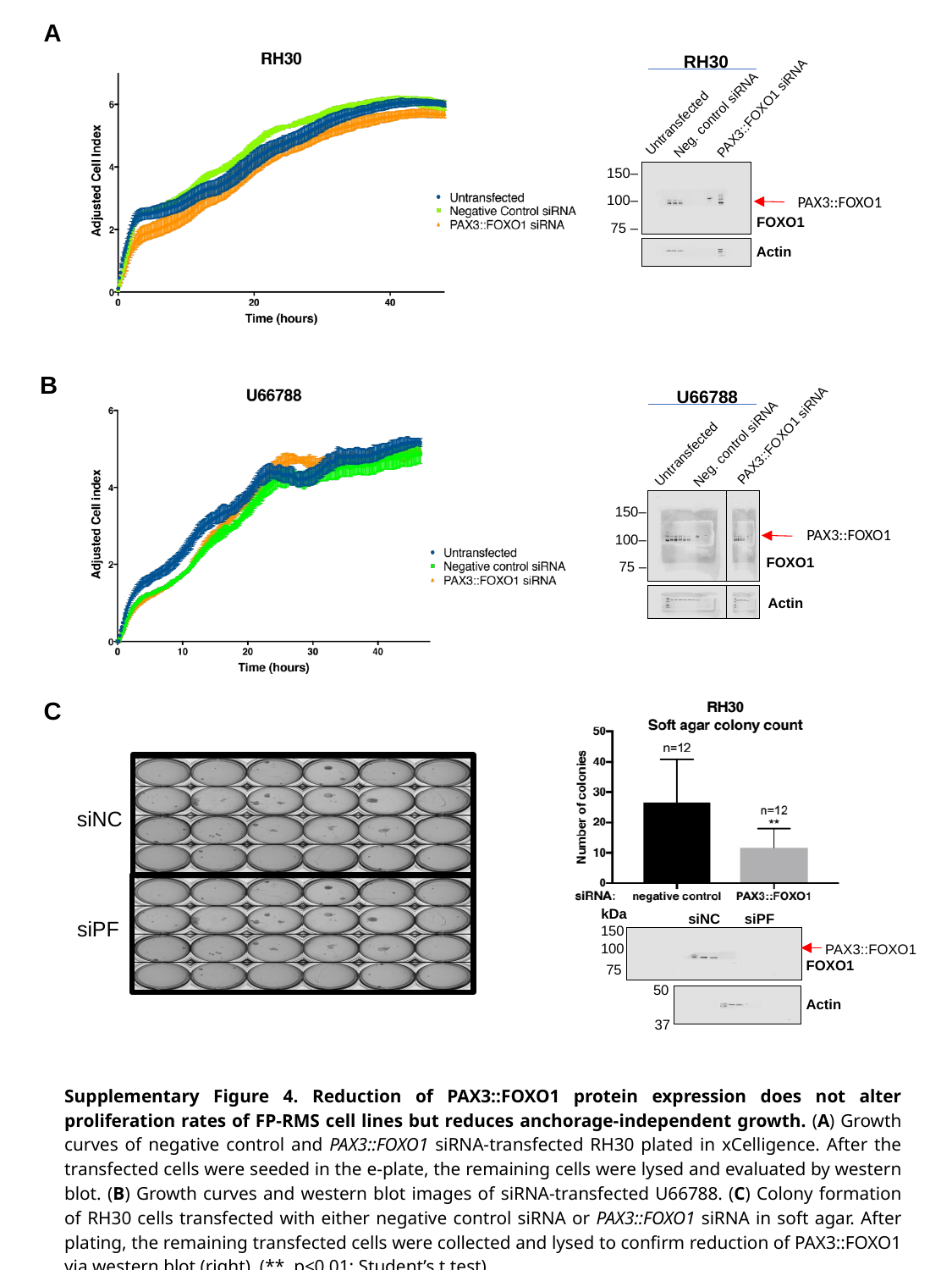

A
RH30
PAX3::FOXO1 siRNA
Neg. control siRNA
Untransfected
150–
100–
 75 –
PAX3::FOXO1
FOXO1
Actin
B
U66788
PAX3::FOXO1 siRNA
Neg. control siRNA
Untransfected
150–
100–
 75 –
PAX3::FOXO1
FOXO1
Actin
C
siNC
kDa
siNC
siPF
siPF
150
100
PAX3::FOXO1
FOXO1
75
50
Actin
37
Supplementary Figure 4. Reduction of PAX3::FOXO1 protein expression does not alter proliferation rates of FP-RMS cell lines but reduces anchorage-independent growth. (A) Growth curves of negative control and PAX3::FOXO1 siRNA-transfected RH30 plated in xCelligence. After the transfected cells were seeded in the e-plate, the remaining cells were lysed and evaluated by western blot. (B) Growth curves and western blot images of siRNA-transfected U66788. (C) Colony formation of RH30 cells transfected with either negative control siRNA or PAX3::FOXO1 siRNA in soft agar. After plating, the remaining transfected cells were collected and lysed to confirm reduction of PAX3::FOXO1 via western blot (right). (**, p<0.01; Student’s t test)
