# Supplementary material for: Piperacetazine Directly Binds to the PAX3::FOXO1 Fusion Protein and Inhibits Its Transcriptional Activity
Source: Cancer Res Commun. 2023 Oct 6;3(10):2030–43. doi: 10.1158/2767-9764.CRC-23-0119 (PMC10557868; doi:10.1158/2767-9764.CRC-23-0119)
Supplement: Supplementary Figure 5 — Piperacetazine is not effective in preventing PAX3::FOXO1- mediated tumorigenesis at the tested dose. [file crc-23-0119-s08.pptx]

## Slide 1
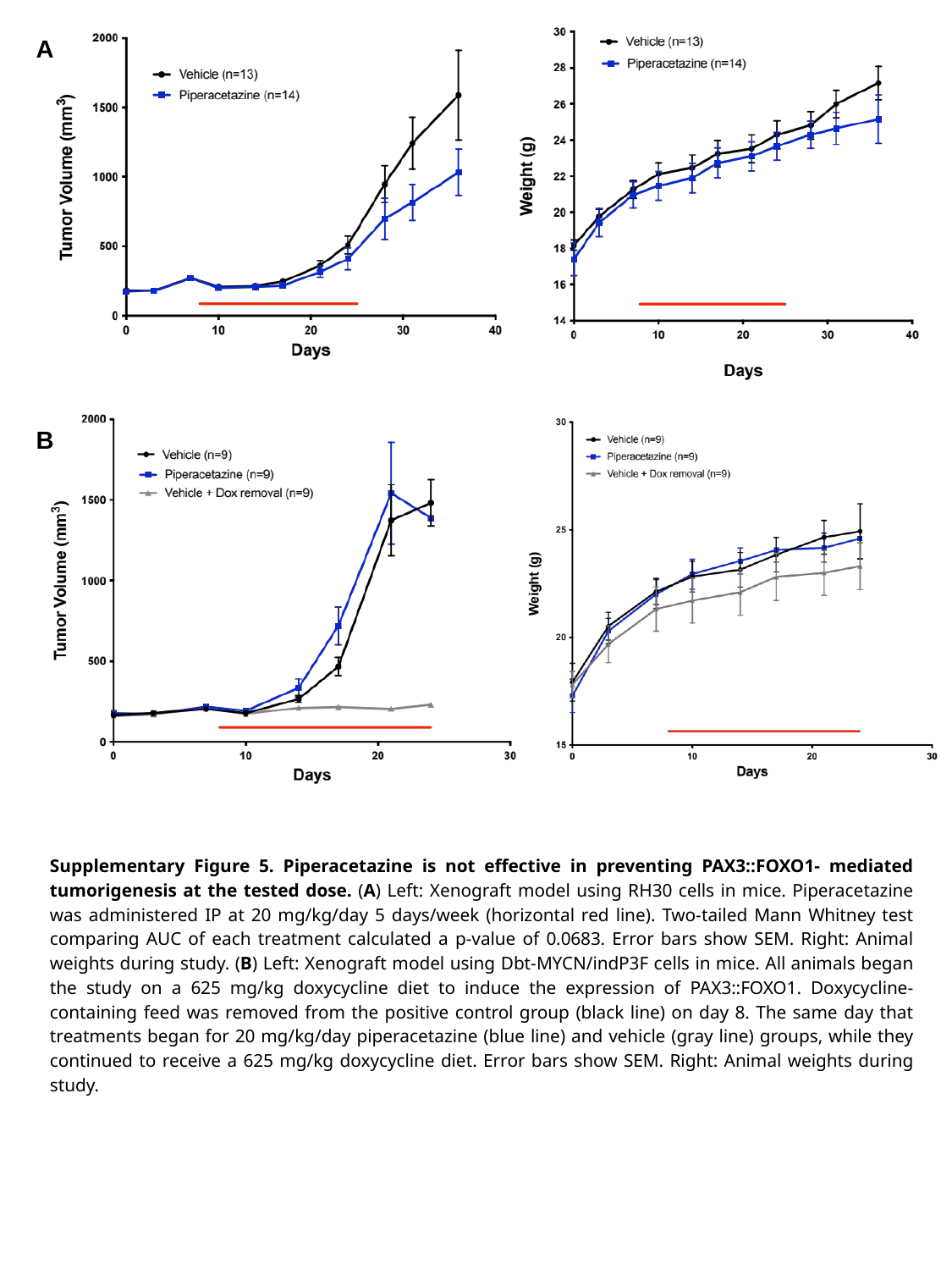

A
B
Supplementary Figure 5. Piperacetazine is not effective in preventing PAX3::FOXO1- mediated tumorigenesis at the tested dose. (A) Left: Xenograft model using RH30 cells in mice. Piperacetazine was administered IP at 20 mg/kg/day 5 days/week (horizontal red line). Two-tailed Mann Whitney test comparing AUC of each treatment calculated a p-value of 0.0683. Error bars show SEM. Right: Animal weights during study. (B) Left: Xenograft model using Dbt-MYCN/indP3F cells in mice. All animals began the study on a 625 mg/kg doxycycline diet to induce the expression of PAX3::FOXO1. Doxycycline-containing feed was removed from the positive control group (black line) on day 8. The same day that treatments began for 20 mg/kg/day piperacetazine (blue line) and vehicle (gray line) groups, while they continued to receive a 625 mg/kg doxycycline diet. Error bars show SEM. Right: Animal weights during study.
