# Supplementary material for: Piperacetazine Directly Binds to the PAX3::FOXO1 Fusion Protein and Inhibits Its Transcriptional Activity
Source: Cancer Res Commun. 2023 Oct 6;3(10):2030–43. doi: 10.1158/2767-9764.CRC-23-0119 (PMC10557868; doi:10.1158/2767-9764.CRC-23-0119)
Supplement: Supplementary Figure 6 — Synergy between piperacetazine and other chemotherapeutic drugs are similar between the RH30 and RD cell lines. [file crc-23-0119-s09.pptx]

## Slide 1
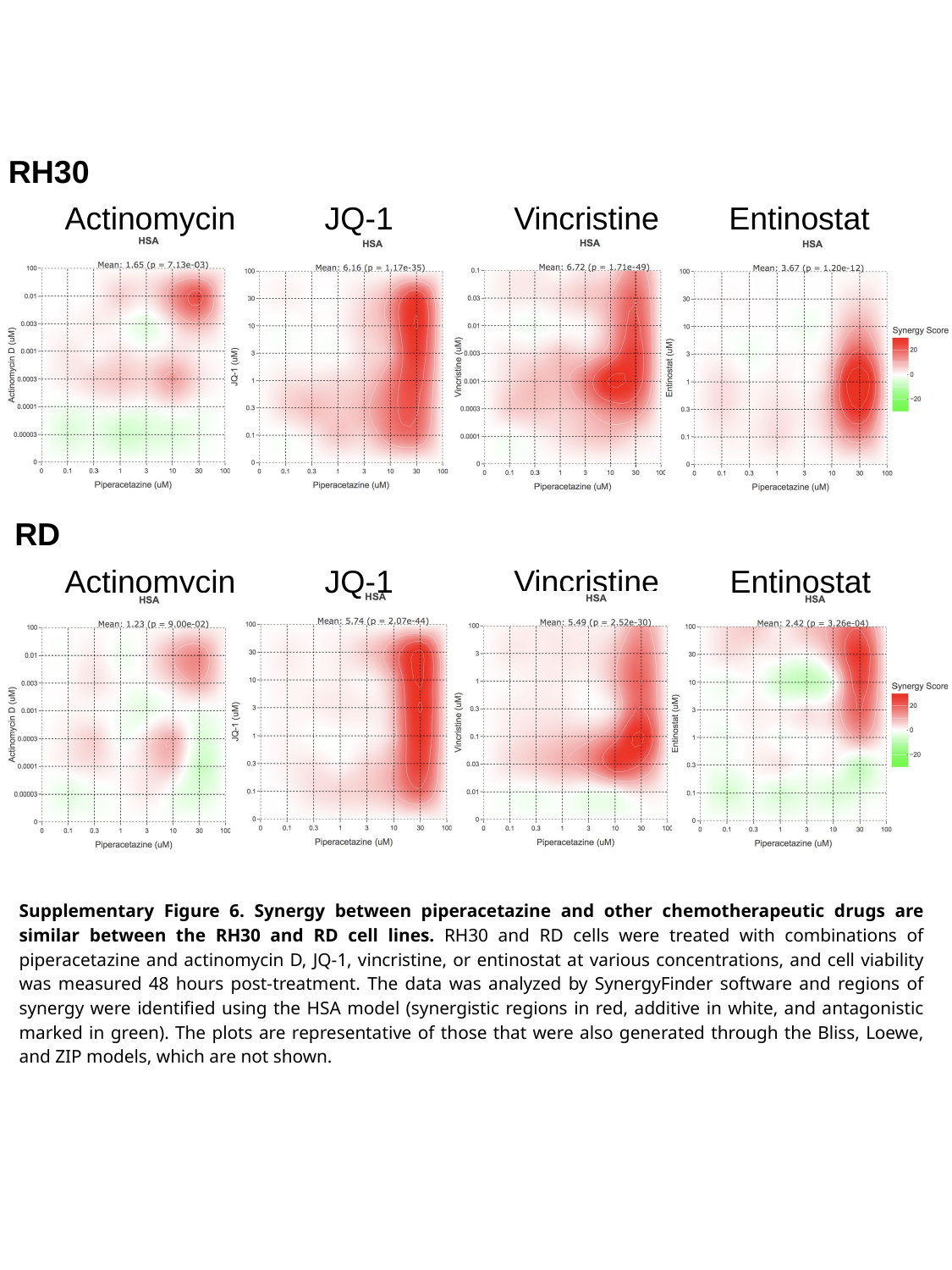

RH30
Actinomycin
JQ-1
Vincristine
Entinostat
RD
Actinomycin
JQ-1
Vincristine
Entinostat
Supplementary Figure 6. Synergy between piperacetazine and other chemotherapeutic drugs are similar between the RH30 and RD cell lines. RH30 and RD cells were treated with combinations of piperacetazine and actinomycin D, JQ-1, vincristine, or entinostat at various concentrations, and cell viability was measured 48 hours post-treatment. The data was analyzed by SynergyFinder software and regions of synergy were identified using the HSA model (synergistic regions in red, additive in white, and antagonistic marked in green). The plots are representative of those that were also generated through the Bliss, Loewe, and ZIP models, which are not shown.
